# Synthesis of Chromone-Related Pyrazole Compounds

**DOI:** 10.3390/molecules22101665

**Published:** 2017-10-05

**Authors:** Clementina M. M. Santos, Vera L. M. Silva, Artur M. S. Silva

**Affiliations:** 1 School of Agriculture, Polytechnic Institute of Bragança, 5300-253 Bragança, Portugal; clems@ipb.pt; 2 Department of Chemistry & QOPNA, University of Aveiro, Campus Universitário de Santiago, 3810-193 Aveiro, Portugal; verasilva@ua.pt

**Keywords:** chromone, dyads, heterocycles, pyrazole, reactivity, review, organic synthesis

## Abstract

Chromones, six-membered oxygen heterocycles, and pyrazoles, five-membered two-adjacent-nitrogen-containing heterocycles, represent two important classes of biologically active compounds. Certain derivatives of these scaffolds play an important role in medicinal chemistry and have been extensively used as versatile building blocks in organic synthesis. In this context, we will discuss the most relevant advances on the chemistry that involves both chromone and pyrazole rings. The methods reviewed include the synthesis of chromone-pyrazole dyads, synthesis of chromone-pyrazole-fused compounds, and chromones as starting materials in the synthesis of 3(5)-(2-hydroxyaryl)pyrazoles, among others. This review will cover the literature on the chromone and pyrazole dual chemistry and their outcomes in the 21st century.

## 1. Introduction

**Figure 1:**
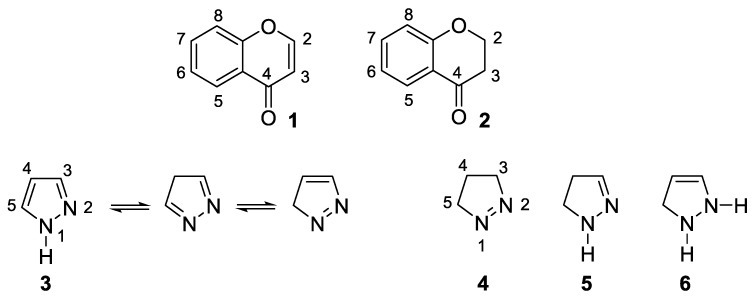
Chemical structures and numbering of compounds **1**–**6**.

4*H*-Benzopyran-4-ones, 4*H*-chromen-4-ones or simply chromones **1** (Figure 1) are six-membered oxygen-containing heterocyclic compounds widespread in Nature. The structural diversity regarding type, number and position of substituents attached to the main core are especially important to the physical, chemical and biological properties of both natural and synthetic derivatives [1,2,3]. Moreover, the chromone moiety is nowadays an active pharmacophore used in varied therapeutic fields in drugs such as cromolyn, nedocromil, diosmin, flavoxate, among others [2,4]. Chromone and its reduced form chromanone (4*H*-chroman-4-one, **2**, Figure 1) are also valuable intermediates in the synthesis of novel bioactive compounds and of new heterocyclic systems [1,5].

Pyrazoles (1*H*-pyrazoles, **3**, Figure 1) are constituted by an aromatic five-membered ring with three carbons and two nitrogen atoms, located at the 1- and 2-positions and are one of the most studied groups of compounds among the azole family [6]. These studies have involved a huge variety of natural and synthetic analogues which have been applied, over the years, in areas such as technology, medicine and agriculture. In fact, drugs such as celecobix, rimonabant and sildenafil are currently used as therapeutic agents [6,7]. *N-*Unsubstituted pyrazoles may present three identical and non-separable tautomers, due to rapid interconversion in solution, and it is usually impossible to unequivocally assign the proton resonances of the pyrazole core in the proton-nuclear magnetic resonance (^1^H-NMR) spectra of these compounds. Three partially reduced forms may also exist: 1-pyrazolines **4**, 2-pyrazolines **5** and 3-pyrazolines **6** (Figure 1) [6,7].

Inspired by this knowledge, research devoted to the synthesis and transformation of both chromone and pyrazole units remain an interesting and challenging topic for organic chemists. In this context, the present review will present and discuss the most relevant developments in the chemistry that involves these two classes of heterocyclic compounds from the year 2000 till the present. The transformations reviewed include: (i) synthesis of chromone-pyrazole dyads using a pyrazole moiety as substituent (via cyclodehydration and oxidative cyclization reactions) and involving pyrazole ring formation (via 1,3-dipolar cycloaddition, condensation of hydrazines with α,β-unsaturated ketones, Knoevenagel reaction and other reactions); (ii) synthesis of chromone-pyrazole-fused compounds through tandem reactions of 3-formylchromones with pyrazole derivatives and other transformations; (iii) condensation reactions of chromones with different hydrazines for the synthesis of 3(5)-(2-hydroxyaryl)pyrazoles and (iv) other reactions involving chromones and pyrazoles not included in the topics described before.

## 2. Synthesis of Chromone-Pyrazole Dyads

### 2.1. Pyrazole as Substituent

#### 2.1.1. Cyclodehydration

**Scheme 1:**
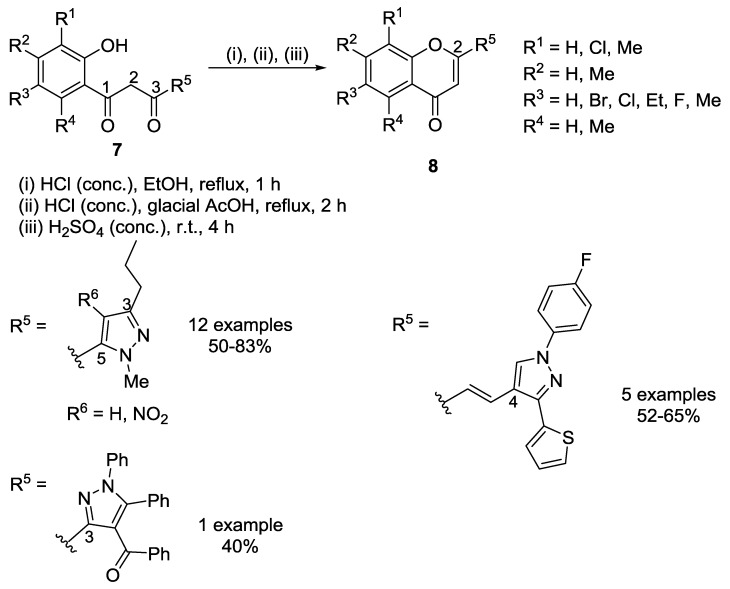
Synthesis of 2-(pyrazol-5/3-yl)chromones and (*E*)-2-[2-(pyrazol-4-yl)vinyl]chromones **8** by cyclodehydration of the appropriate 3-substituted-1-(2-hydroxyaryl)propane-1,3-diones **7** [8,9,10]. Abbreviation: r.t., room temperature.

There has been particular interest in the synthesis of flavonoids with a pyrazole ring at the C-2 position to discover new and more potent biological pharmacophores. The cyclodehydration of 3-pyrazolyl-substituted-1-(2-hydroxyaryl)propane-1,3-diones **7** is known to afford 2-pyrazolyl-chromones **8**, as the main reaction products. These chromone-pyrazole dyads **8** can have the pyrazole substituent linked by the carbon atoms C-5, C-4 or C-3 at carbon C-2 of the chromone scaffold, depending on the pyrazole used as substituent at the 3-position of the starting material **7**. In 2004, Gill and coworkers described the synthesis of 2-(1,3-/1,3,5-di/tri-substituted-pyrazol-5-yl)chromones [8], which were obtained in moderate to good yields (50–83%) by cyclization of the appropriate 1-(2-hydroxyaryl)propane-1,3-diones **7** in refluxing ethanol with a catalytic amount of hydrochloric acid (step (i), Scheme 1). Only two of the nine derivatives of **7** presented moderate phosphodiesterase IV enzyme inhibition activity [8]. Following a slightly different procedure, using glacial acetic acid instead of ethanol, along with a catalytic amount of hydrochloric acid, Karale and coworkers prepared 2-{(*E*)-2-[1-(4-fluorophenyl)-3-(thiophen-2-yl)-1*H*-pyrazol-4-yl]vinyl}chromones [9], in moderate yields (52–65%) (step (ii), Scheme 1). A single example of a 2-(1,4,5-trisubstituted-pyrazol-3-yl)chromone was reported by Menges and coworkers [10]. This compound was obtained in 40% yield by cyclodehydration of the appropriate 1-(2-hydroxyaryl)propane-1,3-dione **7** in concentrated sulfuric acid at room temperature (step (iii), Scheme 1).

**Scheme 2:**
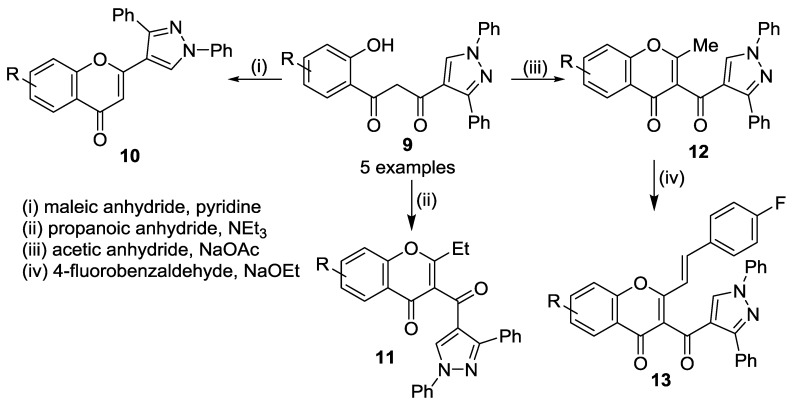
Synthesis of 2-(pyrazol-4-yl)chromones **10** and 3-(pyrazole-4-carbonyl)chromones **11**, **12** and **13** [11].

The cyclodehydration of 1-(2-hydroxyaryl)propane-1,3-diones **9** with maleic anhydride in pyridine gave 2-(1,3-diphenyl-1*H*-pyrazol-4-yl)chromones **10** (Scheme 2) [11]. The same compounds **9** on treatment with propanoic anhydride/triethyl amine undergo cyclization and afforded 2-ethyl-3-(1,3-diphenyl-1*H*-pyrazole-4-carbonyl)chromones **11** whereas the cyclization of **9** in acetic anhydride/sodium acetate gave 2-methyl-3-(1,3-diphenyl-1*H*-pyrazole-4-carbonyl)chromones **12** (Scheme 2) [11]. Further reaction of compound **12** with 4-fluorobenzaldehyde in presence of sodium ethoxide gave 3-(1,3-diphenyl-1*H*-pyrazole-4-carbonyl)-2-[2-(4-fluorophenyl)vinyl]chromones **13** (Scheme 2) [11].

#### 2.1.2. Oxidative Cyclization

**Scheme 3:**
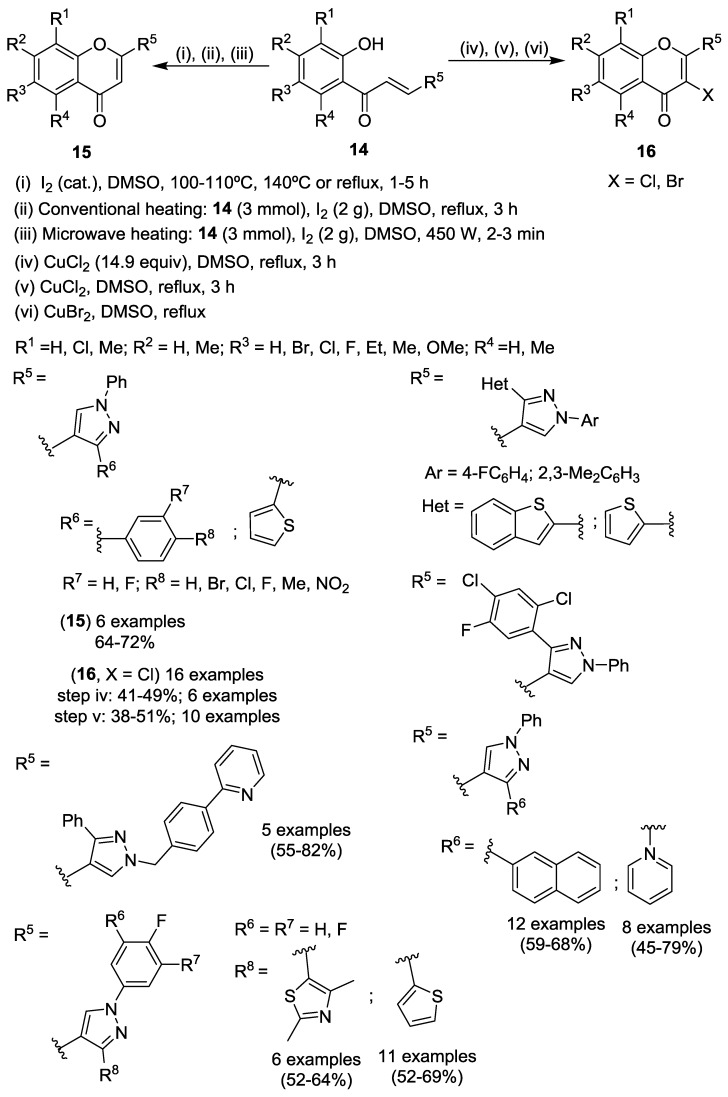
Synthesis of 2-(pyrazol-4-yl)chromones **15** and 3-halo-2-(pyrazol-4-yl)chromones **16** by oxidative cyclization of appropriate 2′-hydroxychalcones **14** [12,13,14,15,16,17,18,19,20,21,22,23,24,25]. Abbreviations: cat., catalytic; DMSO, dimethyl sulfoxide ; equiv, molar equivalent.

The oxidative cyclization of 2′-hydroxychalcone-type compounds **14** is known to afford 2-(pyrazol-4-yl)chromones **15** or 3-halo-2-(pyrazol-4-yl)chromones **16**, depending on the reaction conditions (Scheme 3).

The substitution at carbon C-2 of the chromone unit can include different pyrazoles linked by the C-4 carbon such as 3-aryl-1-phenyl-1*H*-pyrazol-4-yl [12,13,14,15], 1-aryl-3-hetero-substituted-1*H*-pyrazol-4-yl [16,17], 3-(2,4-dichloro-5-fluorophenyl)-1-phenyl-1*H*-pyrazol-4-yl [18], 1-phenyl-3-substituted-1*H*-pyrazol-4-yl [19,20,21], 3-phenyl-l-[4-(pyridin-2-yl)benzyl]-l*H*-pyrazol-4-yl [22] and 1-aryl-3-heteraryl-1*H*-pyrazol-4-yl [9,23,24,25]. Regarding the reaction conditions, 2-(pyrazol-4-yl)chromones **15** were prepared, in most cases, by heating the appropriate 2′-hydroxychalcones in dimethyl sulfoxide (DMSO) in the presence of a catalytic amount of iodine, using conventional heating conditions (steps (i) and (ii), Scheme 3); the temperature of the reaction being 100–110 °C [9,22,23,24], 140 °C [9,12,13,14,22,23,24], or reflux [16,17,18,19,20,21]. A wide range of these 2-(pyrazol-4-yl)chromones **15** were screened for their antibacterial and antifungal potential, presenting from moderate to good activity [12,14,17,18,20,21,22,23,24,25]. The oxidative cyclization of l-(2-hydroxyaryl)-3-{3-phenyl-l-[4-(pyridin-2-yl)benzyl]-l*H*-pyrazol-4-yl}prop-2-en-l-ones with DMSO/I_2_ was performed in both, conventional and microwave heating conditions (steps (ii) and (iii), Scheme 3), affording 2-{3-phenyl-l-[4-(pyridin-2-yl)benzyl]-l*H*-pyrazol-4-yl}-substituted-chromones in 55–62% and 69–82% yield, respectively [22]. The use of microwave heating led to the shortening of the reaction time from 3 h to 2–3 min and to the improvement of the reaction yield.

When heating 2′-hydroxychalcone-type compounds **14** in DMSO in the presence of copper halides such as CuCl_2_ or CuBr_2_, 3-halo-2-(pyrazol-4-yl)chromones **16** were obtained (steps (iv), (v) and (vi), Scheme 3). Thus, 2-(3-aryl/heteryl-1-phenyl-1*H*-pyrazol-4-yl)-3-chlorochromones [12,13,14,15] and 2-[3-(1-benzothiophen-2-yl)-1-(4-fluorophenyl)-1*H*-pyrazol-4-yl]-3-chlorochromone [17] were prepared by oxidative cyclization of the appropriate 2′-hydroxychalcones in DMSO/CuCl_2_ (steps (iv), (v), Scheme 3). Similarly, 3-bromo-2-[3-(2,4-dichloro-5-fluorophenyl)-1-phenyl-1*H*-pyrazol-4-yl]chromone [18] was prepared by treatment of the appropriate 2′-hydroxychalcone with DMSO/CuBr_2_ (step (vi), Scheme 3).

**Scheme 4:**
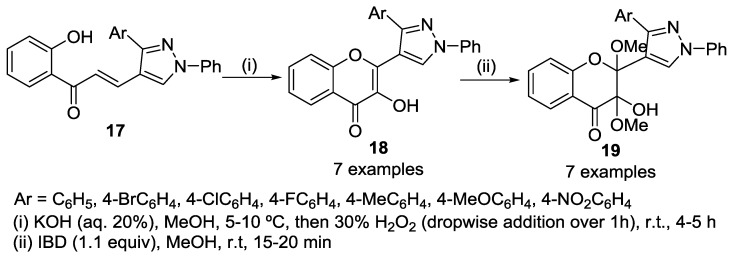
Synthesis of 2-(3-aryl-1-phenyl-1*H*-pyrazol-4-yl)-3-hydroxychromones **18** by Algar-Flynn-Oyamada (AFO) reaction of 2′-hydroxychalcone-type compounds **17** and their oxidation to 2-(3-aryl-1-phenyl-1*H*-pyrazol-4-yl)-3-hydroxy-2,3-dimethoxychromanones **19** [26,27]. Abbreviation: IBD, iodobenzene diacetate.

Prakash and coworkers reported the synthesis of seven new 2-(3-aryl-1-phenyl-1*H*-pyrazol-4-yl)-3-hydroxychromones **18** which were obtained in good yields (52–61%) by the oxidation of 2’-hydroxychalcone-type compounds **17** in an Algar-Flynn-Oyamada (AFO) reaction with hydrogen peroxide (H_2_O_2_) in KOH-MeOH (Scheme 4) [26]. Five derivatives of **18** demonstrated noticeably higher antifungal activity than commercial antifungal compound actidione (cycloheximide) against *Helminthosporium* species, *Fusarium oxysporum* and *Alternaria alternate*, three phytopathogenic fungi. It is noteworthy the effect of the substituents of aryl ring of pyrazole moiety (at 3-position) in compounds **18**; the replacement of the proton of this aryl ring with electron-donating groups led to the increase of the antifungal activity while the opposite effect occurs with electron-withdrawing groups [26]. In 2009, the same author reported the oxidation of 2-(3-aryl-1-phenyl-1*H*-pyrazol-4-yl)-3-hydroxychromones **18** with iodobenzene diacetate in methanol that afforded new 2-(3-aryl-1-phenyl-1*H*-pyrazol-4-yl)-3-hydroxy-2,3-dimethoxychromanones **19** in good yields (72–80%) (Scheme 4) [27]. Three of these compounds **19** showed very good antibacterial activity against both Gram-positive and Gram-negative bacteria, with values comparable with the commercial antibiotics linezolid, cefaclor and cefuroxime axetial [27].

### 2.2. Pyrazole Ring Formation

#### 2.2.1. 1,3-Dipolar Cycloaddition

**Scheme 5:**
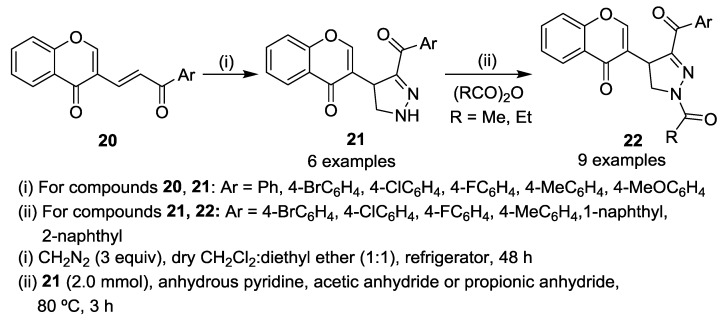
1,3-Dipolar cycloaddition of 3-(3-aryl-3-oxoprop-1-en-1-yl)chromones **20** with diazomethane and further *N*-acetylation of the formed 3-(3-aroyl-2-pyrazolin-4-yl)chromones **21** [28].

A common procedure for the synthesis of pyrazolines (dihydropyrazoles) is through 1,3-dipolar cycloaddition reaction of diazoalkanes to carbon–carbon double bonds. Lévai and Jekő reported the synthesis of 3-(3-aroyl-2-pyrazolin-4-yl)chromones **21** by 1,3-dipolar cycloaddition reaction of 3-(3-aryl-3-oxoprop-1-en-1-yl)chromones **20** with diazomethane in a 1:1 mixture of anhydrous dichloromethane and diethyl ether at ca. 0 °C for 48 h (Scheme 5) [28]. 3-(3-Aroyl-2-pyrazolin-4-yl)chromones **21** were obtained as sole isolable product in 61–89% yield. The reaction was completely regioselective affording 1-pyrazolines which rearrange into 2-pyrazolines, where the methylene moiety of the diazomethane is attached to the β-carbon atom of the α,β-enone. Some of these 2-pyrazolines were further *N*-acylated with a mixture of anhydrous pyridine and acetic anhydride or propionic anhydride at 80 °C for 3 h. The expected *N*-acylated derivatives **22** were obtained in 63–84% yield and neither the 2-pyrazoline nor the chromone ring suffered any rearrangement under these acylating conditions (Scheme 5) [28].

**Scheme 6:**
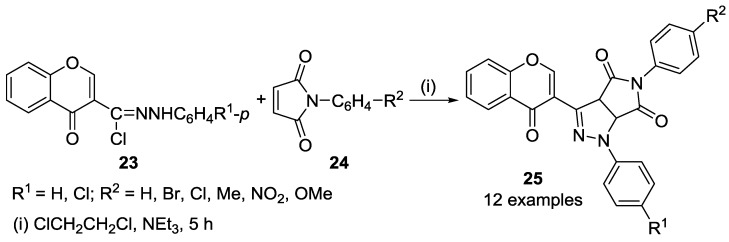
Synthesis of 3-(chromon-3-yl)-3a,6a-dihydro-4,6-dioxopyrrolo[3,4-*d*]pyrazoles **25** by 1,3-dipolar cycloaddition reaction [29].

**Scheme 7:**
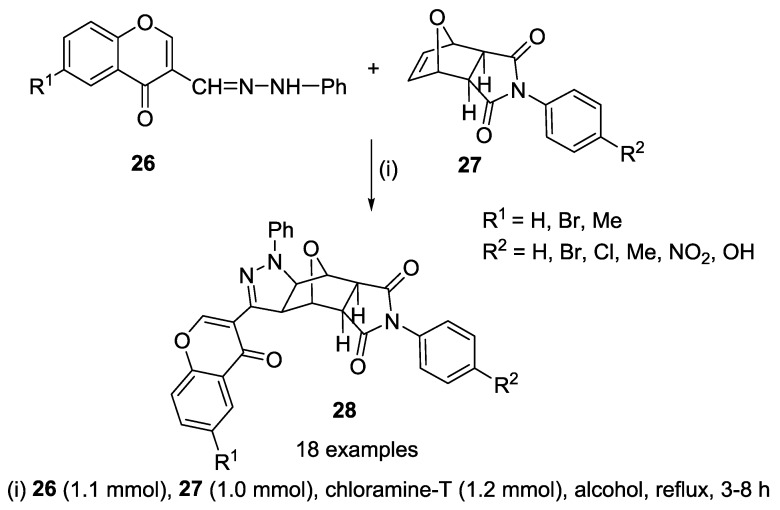
Synthesis of pyrazole-linked norcantharidin derivatives of substituted chromones **28** [30].

The 1,3-dipolar cycloaddition reactions of nitrile imines **23**, prepared in situ from *N*-phenylchromone-3-carbohydrazonoyl chloride, with *N*-arylmaleimides **24** afforded a series of 3-(chromon-3-yl)-3a,6a-dihydro-4,6-dioxopyrrolo[3,4-*d*]pyrazoles **25** in moderate yields (43–55%) (Scheme 6) [29]. A similar reaction occurs between norcantharidin derivatives of substituted aromatic amines **27** with the 3-formylchromone phenylhydrazones **26** in the presence of chloramine-T as catalyst to provide novel pyrazole-linked norcantharidin derivatives substituted at chromone ring **28** (Scheme 7) [30].

**Scheme 8:**
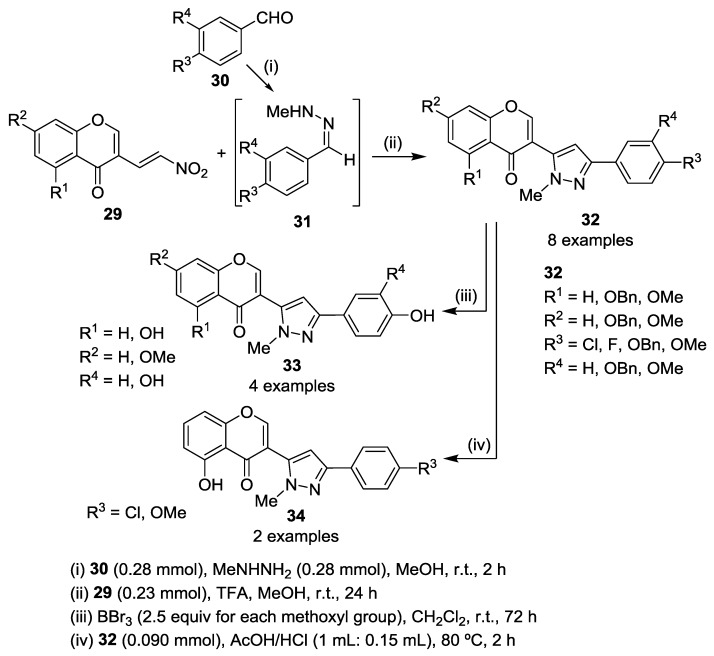
Synthesis of 3-(3-aryl-1-methyl-1*H*-pyrazol-5-yl)chromones **32** by cycloaddition of 3-(2-nitrovinyl)chromones **29** with in situ generated *N*-methylhydrazones **31**, and their hydroxylated derivatives **33** and **34** [31].

The cycloaddition reaction of 3-(2-nitrovinyl)chromones **29** with the in situ prepared *N*-methylhydrazones **31** in methanol in the presence of catalytic amounts of trifluoroacetic acid (TFA) gave the corresponding 3-(3-aryl-1-methyl-1*H*-pyrazol-5-yl)chromones **32** in good yields (55–69%) (Scheme 8) [31].

The hydroxylated 3-(pyrazol-5-yl)chromones **33** and **34** were obtained using different reaction conditions; compounds **33** were isolated in good yields (69–75%) by treating the corresponding methoxy-substituted derivatives with BBr_3_ in dichloromethane and compounds **34** were obtained in excellent yields (97–98%) by treating the corresponding benzyloxy-substituted derivatives with a mixture of acetic acid/hydrochloric acid at 80 °C (Scheme 8) [31]. Among the synthesized compounds, a derivative containing a catechol moiety demonstrated both 1,1-diphenyl-2-picrylhydrazyl (DPPH) free radical scavenging activity and α-glucosidase inhibitory activity [31].

**Scheme 9:**
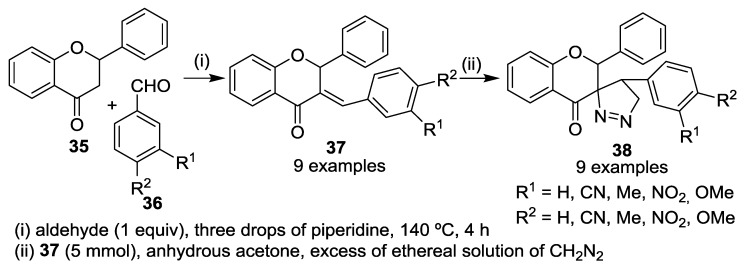
Synthesis of 4′-aryl-2-phenylspiro(1-pyrazoline-3′,3-chromanones) **38** via reaction of 3-benzylidenoflavanones **37** with diazomethane [32].

The synthesis of 3-benzylidenoflavanones **37** via condensation of flavanone **35** (2-phenylchromanone) with aromatic aldehydes **36**, in the presence of a catalytic amount of piperidine and their further reaction with diazomethane led to the formation of a pyrazoline ring condensed at carbon C-3 of the pyrone ring (Scheme 9) [32]. These spiropyrazolines **38** were the only product confirmed by high performance liquid chromatography (HPLC) obtained in good yields from the reaction of **37** with diazomethane [32]. The cytotoxic effect of the nine spiropyrazolines **38** was determined on two human leukaemia cell lines (HL-60 and NALM-6) and melanoma (WM-115) cells, as well as on normal human umbilical vein endothelial cells (HUVEC). The highest cytotoxicity was observed for the *para*-methoxy-derivative, with an half maximal inhibitory concentration (IC_50_) < 10 mM for all three cancer cell lines, with five to twelve-fold lower sensitivity against normal cells (HUVEC) [32].

**Scheme 10:**
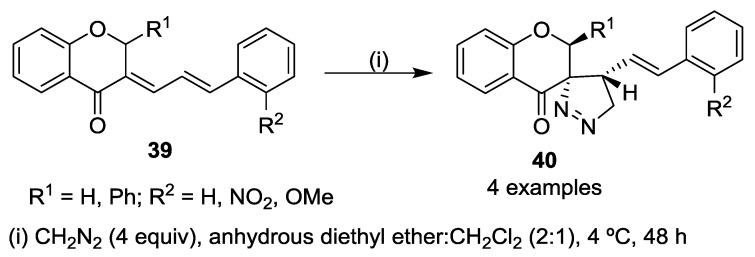
Synthesis of *trans*-4′-styrylspiro(1-pyrazolines-3′,3-chromanones) **40** by the reaction of exocyclic α,β,γ,δ-diunsaturated ketones **39** with diazomethane [33].

1,3-Dipolar cycloaddition reaction of (*E*,*E*)-3-(3-arylallylidene)chromanone **39** with diazomethane (generated in situ by the reaction of *N*-nitroso-*N*-methylurea with potassium hydroxide) at 4 °C afforded *trans*-4′-styrylspiro(1-pyrazolines-3′,3-chromanones) **40** in good yields (72–86%) and in a regioselective and stereospecific way (Scheme 10) [33]. The stereospecific formation of these 1-pyrazolines was explained based on a one-step 1,3-dipolar cycloaddition reaction of diazomethane to the less hindered side of the α,β-double bond of the unsaturated ketones [33].

#### 2.2.2. Condensation of Hydrazines with α,β-Unsaturated Ketones

**Scheme 11:**
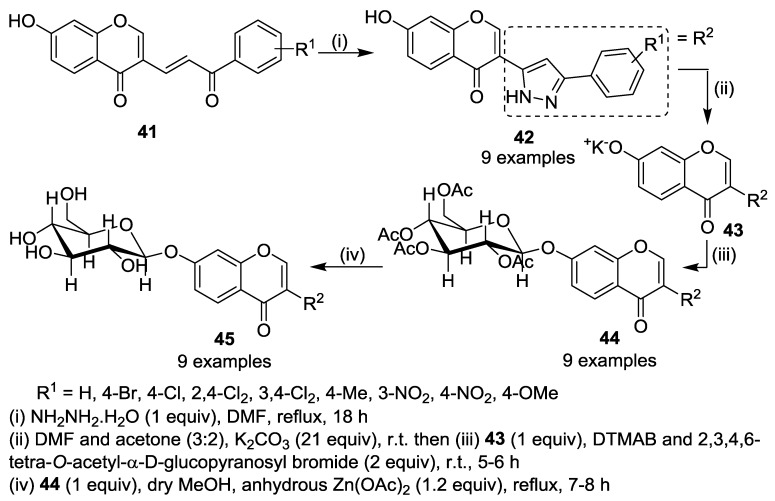
Synthesis of 3-(3-aryl-1*H*-pyrazol-5-yl)-7-β-d-glucopyranosyloxychromones **45** by reaction of 3-(3-aryl-3-oxoprop-1-en-1-yl)-7-hydroxychromones **41** with hydrazine hydrate in DMF followed by *O*-glycosylation and deacetylation [34]. Abbreviations: DMF, dimethylformamide; DTMAB, dodecyltrimethylammonium bromide.

In 2010, Hatzade and coworkers described a convenient procedure for the conversion of 3-(3-aryl-3-oxoprop-1-en-1-yl)-7-hydroxychromones **41** into 3-(3-aryl-1*H*-pyrazol-5-yl)-7-hydroxychromones **42** in 45–67% yield by reaction with hydrazine hydrate in aprotic solvent like dimethylformamide (DMF) (Scheme 11) [34]. Further *O-*glycosylation of **42** (via its potassium salt **43**) was carried out under anhydrous conditions using 2,3,4,6-tetra-*O*-acetyl-α-d-glucopyranosyl bromide as glycosyl donor in the presence of dodecyltrimethylammonium bromide (DTMAB) as a phase transfer catalyst. The reaction was carried out using anhydrous K_2_CO_3_ in a 3:2 mixture of DMF and acetone affording the 3-(3-aryl-1*H*-pyrazol-5-yl)-7-(2,3,4,6-tetra-*O*-acetyl-β-d-glucopyranosyloxy)chromones **44** with high regio- and diastereoselectivity and improved overall yields (78–95%). Deacetylation of **44** with anhydrous zinc acetate in methanol gave the 3-(3-aryl-1*H*-pyrazol-5-yl)-7-β-d-glucopyranosyloxychromones **45** in 70–85% yield (Scheme 11) [34]. Compounds **42** and their aglycones **45** were tested for their in vitro antibacterial activity against *Escherichia coli*, *Klebsiella aerogenes*, *Staphylococcus aureus* and *Bacillus subtilis*; antifungal activity against *Aspergillus niger* and *Candida albicans* fungi as well as DPPH radical scavenging activity. Generally, derivatives **45** showed greater pharmacological activity than the precursor aglycones **42**, being promising antimicrobial and antioxidant pharmacophores [34].

**Scheme 12:**
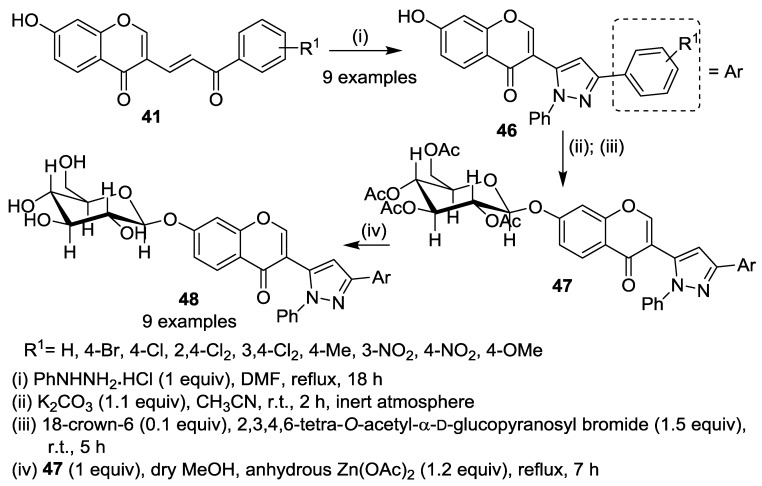
Synthesis of 3-(3-aryl-1-phenyl-1*H*-pyrazol-5-yl)-7-β-d-glucopyranosyloxychromones **48** by reaction of 3-(3-aryl-3-oxoprop-1-en-1-yl)-7-hydroxychromones **41** with phenylhydrazine hydrochloride in DMF followed by *O*-glycosylation and deacetylation [35,36].

Years later, the same group reported computational evaluation using the Petra/Osiris/Molinspiration approach and the experimental verification of novel 3-(3-aryl-1-phenyl-1*H*-pyrazol-5-yl)-7-hydroxychromones **46** and their *O*-β-d-glucopyranosides **48** for their antimicrobial and antioxidant activity [35]. The evaluated compounds **46** were prepared in 45–61% yield from 3-(3-aryl-3-oxoprop-1-en-1-yl)-7-hydroxychromones **41** with phenylhydrazine hydrochloride in DMF [36]. The *O*-glycosylation of **46** with 2,3,4,6-tetra-*O*-acetyl-α-d-glucopyranosyl bromide was achieved in good yields (65–76%) and subsequent deacetylation using the conditions reported by Ingle and Hatzade [35,36] gave 3-(3-aryl-1-phenyl-1*H*-pyrazol-5-yl)-7-β-d-glucopyranosyloxychromones **48** in 69–90% yield (Scheme 12). Based on the above mentioned chemoinformatic studies the authors concluded that the introduction of appropriate di-substituted pyrazole ring into position 3 of chromone ring enhanced antibacterial activities of compounds **46** and **48** [35]. Once more, compounds containing the glucoside unit **48** proved to be more effective antimicrobial and antioxidant agents than the corresponding aglycons **46** [36].

**Scheme 13:**
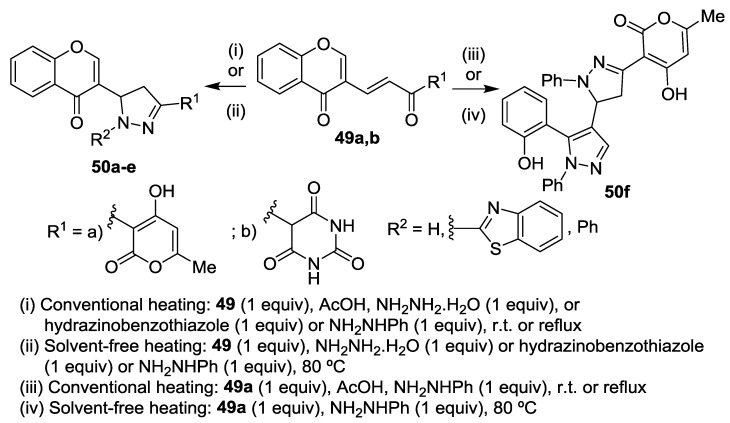
Synthesis of 3-(1,3-disubstituted-2-pyrazolin-5-yl)chromones **50a**–**e** and of a pyrazole-2-pyrazoline **50f** by reaction of 3-(3-substituted-3-oxoprop-1-en-1-yl)chromones **49a**,**b** with different hydrazines [37].

Siddiqui and coworkers reported the reaction of 3-(3-substituted-3-oxoprop-1-en-1-yl)chromones **49** with different hydrazines in conventional heating conditions, using acetic acid as solvent, and in solvent-free heating conditions (Scheme 13) [37].

Both methods gave the expected 3-(1,3-disubstituted-2-pyrazolin-5-yl)chromones **50** from reaction with hydrazine hydrate, hydrazinobenzothiazole and phenylhydrazine with similar results (yields were not presented in the original manuscript). However, the reaction of **49a** with phenylhydrazine did not afford the expected 2-pyrazoline; instead a pyrazole-2-pyrazoline **50f** was obtained due to the reaction of both α,β-unsaturated carbonyl systems, one of them involving also a pyrone ring opening (this mechanism will be discussed in Section 4) in conventional and thermal solvent-free conditions. The synthesized compounds **50,** demonstrated moderate to good antimicrobial activity, which seemed to be dependent on the nature of the heterocyclic moieties. Moreover, although the tested compounds were more active against fungi than bacteria, none of them exceeded the activity of the commercial drugs ciprofloxacin and griseofulvin [37].

#### 2.2.3. Knoevenagel Reaction

**Scheme 14:**
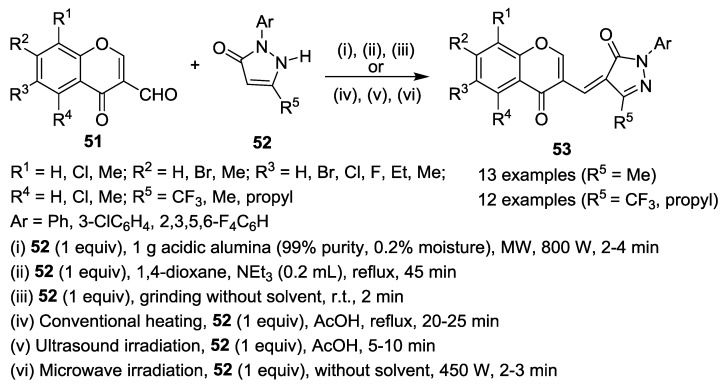
Synthesis of (*E*)-3-[(3-substituted-1-aryl-5-oxo-2-pyrazolin-4-ylidene)methyl]chromones **53** by reaction of 3-formylchromones **51** with 3-substituted-1-aryl-3-pyrazolin-5-ones **52** [38,39,40,41,42,43,44,45,46].

(*E*)-3-[(2-Pyrazolin-/pyrazolidin-4-ylidene)methyl]chromones can be obtained in a very straightforward way through Knoevenagel condensation of 3-formylchromones with appropriate pyrazolin-5-ones [38,39,40,41,42,43,44,45,46] and pyrazolidine-3,5-diones [47], respectively. The 3-formylchromones have three active sites; the chromone carbonyl group, carbon 2 and the formyl group. Of these, the formyl group has the highest reactivity towards active methylene compounds, such as the above mentioned pyrazolin-5-ones and pyrazolidine-3,5-diones. Several examples of this type of condensation reactions have been reported in the literature. In 2002, Shingare and coworkers reported the Knoevenagel condensation of various 3-formylchromones **51** with 3-methyl-1-phenyl-3-pyrazolin-5-one **52** under microwave (MW) irradiation, with alumina support under solvent-free conditions, to obtain (*E*)-3-[(3-methyl-5-oxo-1-phenyl-2-pyrazolin-4-ylidene)methyl]chromones **53** in 59–87% yield, after only 2–4 min of reaction (step (i), Scheme 14) [38]. The efficacy of this method was compared with the same reaction in refluxing 1,4-dioxane using a catalytic amount of triethylamine (step (ii), Scheme 14). The latter method required 45 min for completion of the reaction and the yields were found to be moderate to high (48–80%). Nuclear Overhauser effect (NOE) experiments confirmed that out of possible two isomers the reaction affords only the isomer depicted in Scheme 14. Later, the same group reported a simple, efficient and environmentally friendly method to synthesize compounds **53** in excellent yields (77–90%) by grinding 3-formylchromones **51** with 3-methyl-1-phenyl-3-pyrazolin-5-one **52** in mortar and pestle at room temperature without solvent. Almost quantitative formation of the product (77–90% yield) was achieved within 2 min (step (iii), Scheme 14) [39]. The Knoevenagel reaction of 3-formylchromones **51** with other 3-substituted-1-phenyl-3-pyrazolin-5-ones **52** (R^5^ = CF_3_, propyl) by conventional and non-conventional methods also gave the corresponding (*E*)-3-[(5-oxo-1-phenyl-3-substituted-2-pyrazolin-4-ylidene)methyl]chromones **53** (steps (iv), (v) and (vi), Scheme 14), which demonstrated mild antibacterial and antifungal activities [40,41]. The authors observed that the reactions were very clean and afforded compounds **53** in high yields when using ultrasounds (72–89%) (step (v), Scheme 14) or microwave irradiation (68–81%) (step (vi), Scheme 14) requiring short time for completion (2–10 min) whereas in conventional heating method the reaction time was 20–25 min and the yields were comparatively poor (56–74%) (step (iv), Scheme 14) [40]. The same type of reaction involving other 3-formylchromones and 3-substituted-1-aryl-2-pyrazolin-5-ones in acetic acid under ultrasound or microwave irradiation in solvent-free conditions afforded also the corresponding (*E*)-3-[(1-aryl-3-methyl-5-oxo-2-pyrazolin-4-ylidene)methyl]chromones **53** (steps (v) and (vi), Scheme 14) [42,43,44,45,46]. Some of these chromone-pyrazole conjugates **53** were screened for their biological potential. Thus, compound **53** (R^1^ = R^2^ = R^3^ = R^4^ = H, R^5^ = Me, Ar = 3-ClC_6_H_4_) exhibited mild growth inhibitory activity (30–40%) in four human tumor cell lines (RPMI-8226, SR, M14 and UO-31) [43]. On the other hand, compound **53** (R^1^ = NO_2_, R^2^ = R^4^ = H, R^3^ = F, R^5^ = Me, Ar = 3-ClC_6_H_4_) was revealed as a leading anti-inflammatory drug, presenting IC_50_ values of 10 μM for both cyclooxygenase-1 and cyclooxygenase-2 enzymes [44]. Among the nine derivatives of **53** (R^5^ = CF_3_ or propyl, Ar = 2,3,5,6-F_4_C_6_H), some of them showed promising antibacterial activity against *Staphylococcus aureus*, *Streptococcus pyogenes*, *Escherichia coli* and *Pseudomonas aeruginosa* and fungal pathogen *Candida albicans*, being none of them active for *Aspergillus niger* and *Aspergillus clavatus* fungal strains [45].

**Scheme 15:**
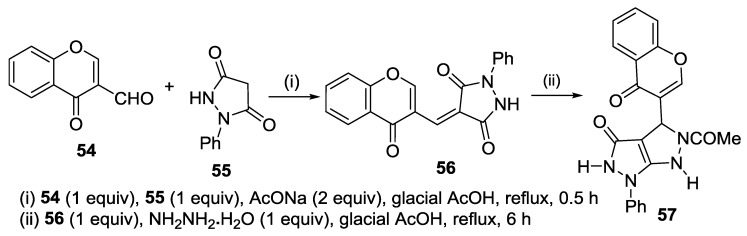
Synthesis of 3-[(3,5-dioxo-1-phenylpyrazolidin-4-ylidene)methyl]chromone **56** and further reaction with an equimolar amount of hydrazine hydrate in glacial acetic acid [47].

The Knoevenagel condensation of 3-formylchromone **54** with 1-phenylpyrazolidine-3,5-dione **55** gave 3-[(3,5-dioxo-1-phenylpyrazolidin-4-ylidene)methyl]chromone **56**. Upon reaction with hydrazine hydrate in 1:1 ratio in glacial acetic acid compound **56** was converted into the corresponding 5-acetyl-4-(chromon-3-yl)-1-phenyl-1,4,5,6-tetrahydropyrazolo-[3,4-*c*]pyrazol-3(2*H*)-one **57** (Scheme 15) [47].

#### 2.2.4. Other Reactions

**Scheme 16:**
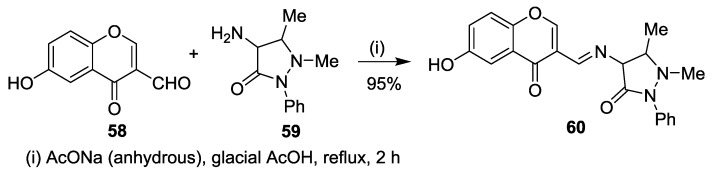
Synthesis of chromone-pyrazolone conjugate **60** [46].

A different chromone-pyrazolone conjugate was reported by Abdel-Rahman who described the condensation reaction of 3-formyl-6-hydroxychromone **58** with 4-amino-3-methyl-1-phenyl-2-pyrazolin-5-one **59** to afford the corresponding imine derivative **60**, in excellent yield (Scheme 16) [46].

**Scheme 17:**
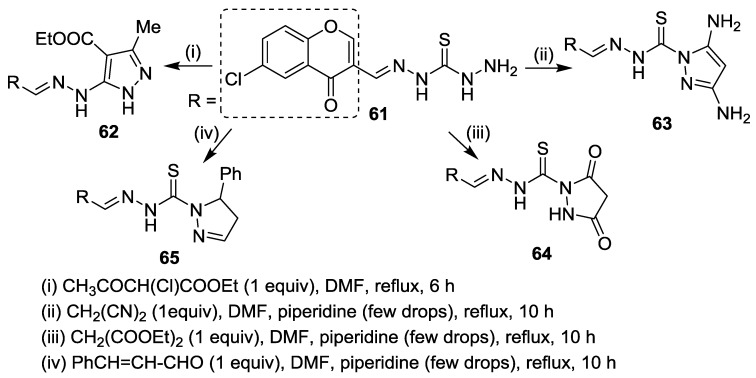
Reactions of 3-formylchromone derived monothiocarbohydrazone **61** with ethyl 2-chloro-acetoacetate, malononitrile, diethyl malonate and cinnamaldehydes [48].

**Scheme 18:**
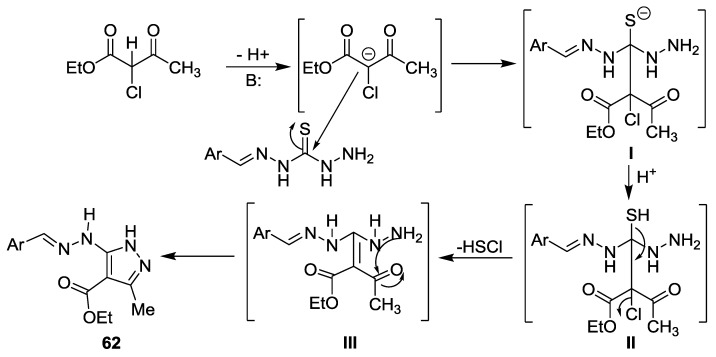
Plausible mechanism for the formation of the unexpected ethyl 5-[2-(6-chlorochromon-3-ylmethylene)hydrazino]-3-methyl-1*H*-pyrazole-4-carboxylate **62** [48].

The reaction of 3-formylchromone derived monothiocarbohydrazone **61** with ethyl 2-chloro-acetoacetate in DMF yielded an unexpected product, the ethyl 5-[2-(6-chlorochromon-3-ylmethylene)hydrazino]-3-methyl-1*H*-pyrazole-4-carboxylate **62** (Scheme 17) [48]. Ali and coworkers also performed the reaction of **61** with malononitrile, diethyl malonate and cinnamaldehyde in DMF and few drops of piperidine which afforded the pyrazole derivatives **63**–**65**, respectively (Scheme 17) [48]. The formation of the unexpected compound **62** was explained by a carbanion attack of ethyl 2-chloroacetoacetate at C=S group of compound **61** to form the intermediate **I** which after accepting a proton and elimination of HSCl give intermediate **III**, which upon cyclocondensation reaction gave compound **62** (Scheme 18) [48]. Only compounds **62** and **64** were screened for their antifungal activity. Thus, compound **64** showed moderate activities against *Alternaria alternata* and *Aspergillus flavipes* and lower activity against *Aspergillus niger* while compound **62** showed moderate activities against these three fungi species [48].

Gill and coworkers reported the reaction of substituted 3-formylchromones with 3-methyl-1-phenyl-1*H*-thieno[2,3-*c*]pyrazole-5-carbohydrazide using acetic acid as catalyst in methanol which gave the corresponding hydrazides in good yield (78–81%) [49]. Two of the four synthesized compounds showed promising antioxidant and anti-inflammatory activities [49].

**Scheme 19:**
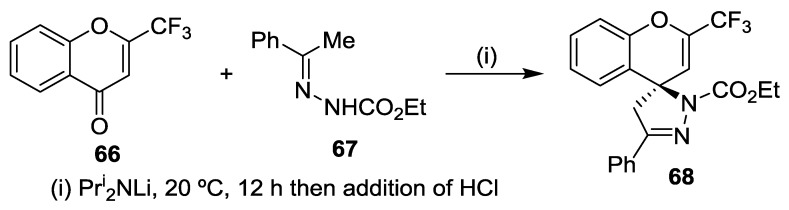
Synthesis of a 2-trifluoromethylchromone-derived spiropyrazoline **68** [50].

Hydrazones of methyl ketones react with chromones at the carbonyl carbon (1,2-addition) to give, upon acidification, spiro(4*H*-chromene-4,5′-pyrazolines). This type of compounds was obtained from the reaction of 2-trifluoromethylchromone **66** with ethyl 2-(1-phenylethylidene)hydrazine-1-carboxylate **67** which afforded spiropyrazoline **68** in 26% yield (Scheme 19) [50].

## 3. Synthesis of Chromone-Fused Pyrazoles

### 3.1. Tandem Reactions of 3-Formylchromones with Pyrazole Derivatives

**Scheme 20:**
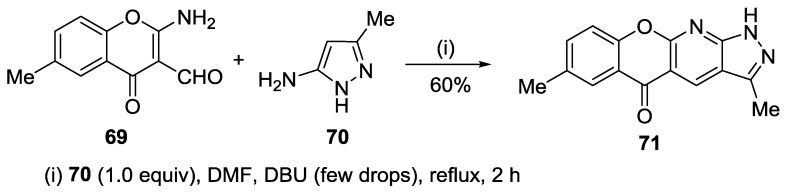
Synthesis of 3,7-dimethylchromeno[2,3-*b*]pyrazolo[4,3-*e*]pyridin-5(1*H*)-one **71** [51]. Abbreviation: DBU, 1,8-diazabicyclo[5.4.0]undec-7-ene.

Structurally diverse chromone-fused pyrazoles can be prepared by tandem reactions of 3-formylchromones with several pyrazole [51] and pyrazolone derivatives [52], including the cycloaddition reaction of 3-formylchromones with pyrazole-*o*-quinodimethane derivatives [53]. The treatment of 3-formylchromone **69** with 5-amino-3-methyl-1*H*-pyrazole **70** in refluxing dimethylformamide/1,8-diazabicyclo[5.4.0]undec-7-ene (DMF/DBU) resulted in 3,7-dimethylchromeno[2,3-*b*]pyrazolo[4,3-*e*]pyridin-5(1*H*)-one **71** in 60% yield (Scheme 20) [51].

**Scheme 21:**
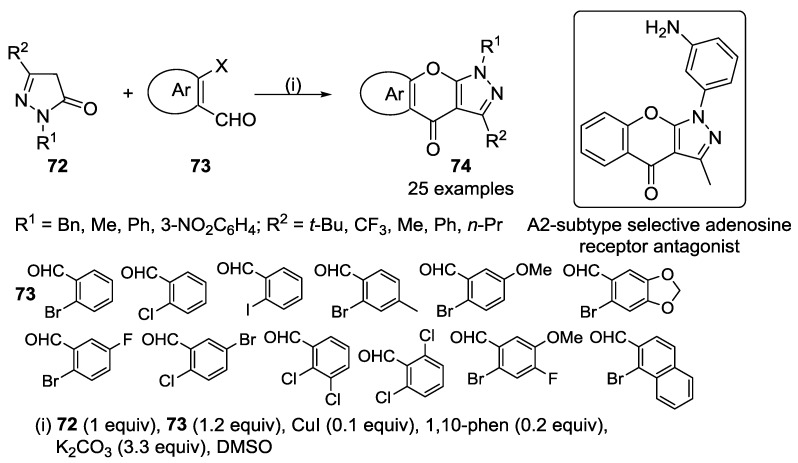
Synthesis of chromone-fused pyrazoles **74** by a tandem *O*-arylation-oxidative coupling reaction between 2-pyrazolin-5-ones **72** and *o*-haloarylaldehydes **73** [52].

Suresh and coworkers reported a straightforward synthesis of chromone-fused pyrazoles **74** by a tandem *O*-arylation-oxidative coupling reaction between 2-pyrazolin-5-ones **72** and *o*-halo-arylaldehydes **73** under aerobic conditions [52]. The reaction was performed using a combination of CuI as catalyst, 1,10-phenantroline as a ligand and K_2_CO_3_ as a base, in DMSO, which proved to be the best combination after a detailed screening of the reaction conditions (Scheme 21). For some derivatives the reaction was scaled-up to a gram scale while maintaining a high yield. The study of the reaction scope showed that 2-bromobenzaldehydes gave better yields of the desired product (74%) when compared to 2-chlorobenzaldehyde (25%) or 2-iodobenzaldehyde (52%). Furthermore, electron-donating groups on 2-bromobenzaldehydes afforded chromone-fused pyrazole derivatives in good yields (51–65%) while electron-withdrawing groups like fluorine gave the product in moderate yield (45%). 2-Bromobenzaldehyde bearing both electron-donating and electron-withdrawing substituents afforded the corresponding product in good yield (64%). A tetracyclic chromone fused pyrazole was obtained in good yield (66%) using 1-bromo-2-naphthaldehyde. Concerning to the substituents of the pyrazolone reagent, different electron-donating and electron-withdrawing substituents were well tolerated furnishing the diversely substituted chromone-fused pyrazole frameworks in moderate to good yields (35–68%). However, the reaction of *N*-Boc-pyrazolone and 2-bromobenzaldehyde did not give the desired product. Likewise the reaction with heteroaromatic aldehydes such as 2-chloronicotinaldehyde was not well succeeded. The synthetic utility of this method was demonstrated with the synthesis of a representative A2-subtype selective adenosine receptor antagonist (Scheme 21) [52].

**Scheme 22:**
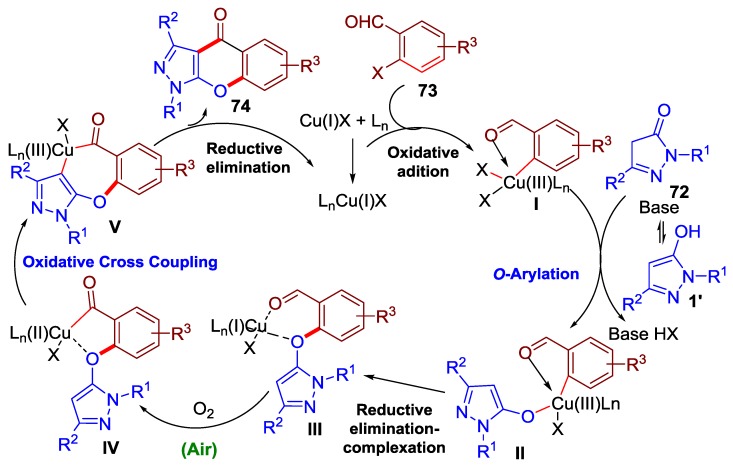
Plausible mechanism of the tandem *O*-arylation-oxidative coupling between 2-pyrazolin-5-ones **72** and *o*-haloarylaldehydes **73** [52].

According to the proposed reaction mechanism (Scheme 22) C–H activation is the key step in this tandem process. The first step is the formation of intermediate **I** by oxidative addition of *o*-haloarylaldehyde **73** with copper catalyst. In the presence of a base, pyrazolone **72** would undergo complexation with the intermediate **I** affording intermediate **II**, which after reductive elimination followed by complexation with the Cu(I) led to the formation of intermediate **III**. Upon oxidative insertion, in the presence of oxygen, intermediate **IV** was obtained and led to the cyclized copper complex **V** that finally undergo cyclization to give the desired product **74** upon reductive elimination [52].

**Scheme 23:**
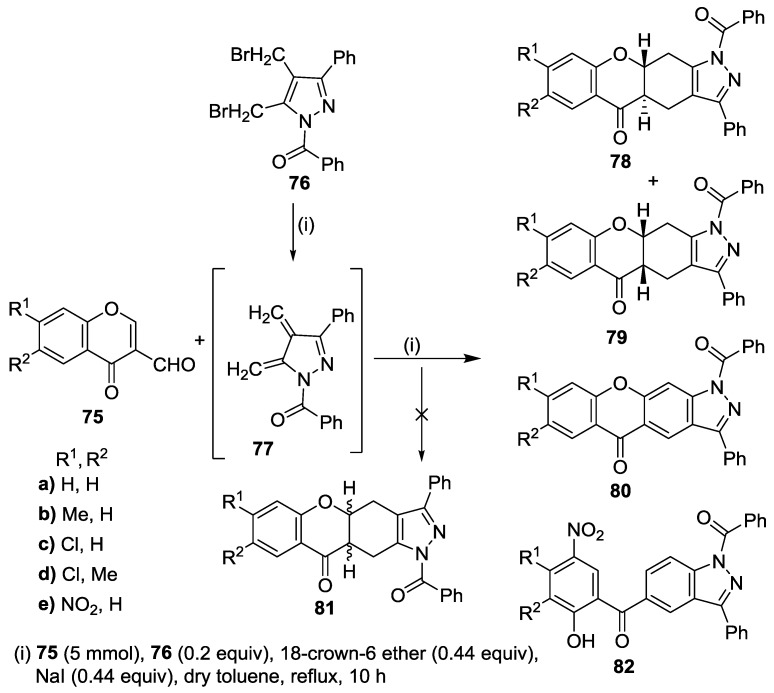
Synthesis of tetrahydrochromeno[3,2-*f*]indazoles **78** and **79** by cycloaddition reaction of 3-formylchromones **75** with pyrazole-*o*-quinodimethane **77** [53].

New fused tetrahydrochromeno[3,2-*f*]indazoles were prepared by incorporating the chromone moiety into the pyrazole nucleus by cycloaddition reaction of chromone **75** with pyrazole-*o*-quinodimethane **77**, generated in situ through reaction of sodium iodide with the appropriate dibromo-derivative **76**. The cycloaddition reaction gave only cycloadducts **78** and **79** along with a small amount of the oxidation product **80**, which, however, was the main reaction product in the case of 3-formylchromone **75a** (Scheme 23) [53]. The reaction is highly regioselective and mixtures of only two diastereomers **78b**–**78e** and **79b**–**79e**, were isolated in moderate yields (20–51%) with the benzoyl group being always on the same side as the pyran oxygen. Although small amounts (less than 2%) of the other possible regioisomers **81** were formed as observed in the ^1^H-NMR spectra of the crude reaction mixture, they were not isolated. In most cases the crude reaction mixture also presented small amounts (2–5% yield) of the corresponding oxidation products **80**. An exception was the reaction with **75a** that afforded the oxidation product **80a** as the main reaction product (35% yield) together with **79a** (20% yield). The authors have postulated that compound **79a** may be formed by the dehydrogenation of the *trans*-bridgehead hydrogens (4a-H and 10a-H). All formed products were prone to deformylation under the reaction conditions. It is also remarkable that opening of the pyran ring was never observed. Yet, upon purification of **78** on preparative thin-layer chromatography (TLC) cleavage of the pyran ring occurred affording the hydroxy derivatives **82** (Scheme 23) [53].

### 3.2. Other Transformations

**Scheme 24:**
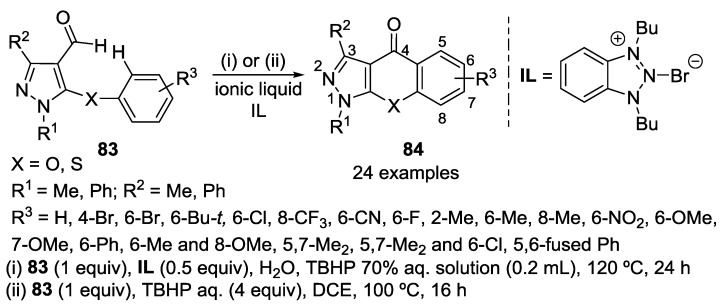
Synthesis of chromeno[2,3-*c*]pyrazol-4(1*H*)-ones **84** via intramolecular cross-dehydrogenative-coupling reaction [54,55]. Abbreviations: IL, ionic liquid; TBHP, *tert*-butyl hydroperoxide; DCE, 1,2-dichloroethane.

Liu and coworkers reported a concise and mild route for the synthesis of chromeno[2,3-*c*]pyrazol-4(1*H*)-ones **84**, in 43–78% yield, by using classical ionic liquids which contained a heterocyclic structure as the promoter, water as a solvent and *tert*-butyl hydroperoxide (TBHP) (70% aqueous solution) as the oxidant without any additives or catalysts, which proceeded through the intramolecular dehydrogenative coupling of the aldehyde C–H bonds and aromatic C–H bonds in 5-aryloxy-4-formyl-1*H*-pyrazoles **83** (Scheme 24) [54]. The ionic liquid was easily recycled and reused with the same efficacies for five cycles and the reaction tolerates diverse functional groups. Substrates bearing either electron-withdrawing or electron-donating groups led to the annulation products in good yields. Aryloxy parts with electron-withdrawing groups are generally more reactive than those with electron-donating groups giving relatively higher yields. Substituents at the *o-*position of the aryloxy group had little influence on the yield but when the substituent was at the *m-*position, the products were obtained as isomers in some cases. Reaction with pyrazoles having 1,3-dimethyl or 1,3-diphenyl groups also proceeded in mild conditions affording the desired products. The reaction was also applicable to the synthesis of a thiochromone which was obtained in good yield (63%). When performed at a gram-scale under the standard conditions the reaction afforded the expected product in 70% isolated yield, while in the model reaction it was obtained in 73% isolated yield. This method constitutes a straightforward and metal-free approach to prepare chromeno[2,3-*c*]pyrazol-4(1*H*)-ones overcoming the limitations found in other methods that require harsh conditions, have limited substrate scope, poor substituent tolerance and give the product in low yield.

Later Singh and coworkers reported a metal/additive-free, TBHP-promoted synthesis of fused chromeno[2,3-*c*]pyrazol-4(1*H*)-ones **84** from 5-aryloxy-4-formyl-3-methyl-1-phenyl-1*H*-pyrazoles **83** also via cross-dehydrogenative coupling of aldehydic C–H bond with arene C–H bond in very good yields (79–85%) (Scheme 24). Similarly, the reaction was found to proceed by a free radical mechanism [55].

**Scheme 25:**
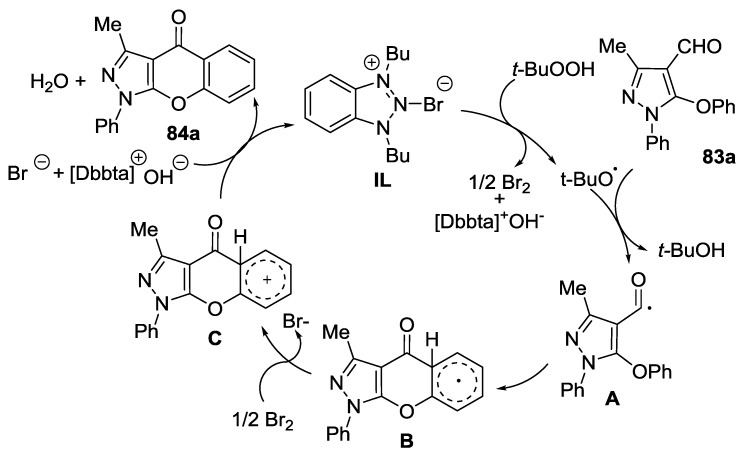
Plausible reaction mechanism for the formation of chromeno[2,3-*c*]pyrazol-4(1*H*)-ones **84** [54].

According to the mechanism proposed by Liu and coworkers (Scheme 25), the reaction proceeds via generation of *t*-butoxyl radicals, promoted by the ionic liquid, which abstracts the aldehyde hydrogen atom to form an acyl radical **A** that adds to the aryloxy unit producing radical **B**. This radical leads to the formation of intermediate **C** via single-electron-transfer process. Then, the previously formed hydroxyl anion acts as the proton abstractor from **C**, providing the annulated product **84a**. The authors proposed another possible mechanism where the acidic proton in **B** is trapped by the hydroxyl anion to give the radical anion intermediate. Formal liberation of an electron from this intermediate eventually leads to the formation of the product **84a** [54].

**Scheme 26:**
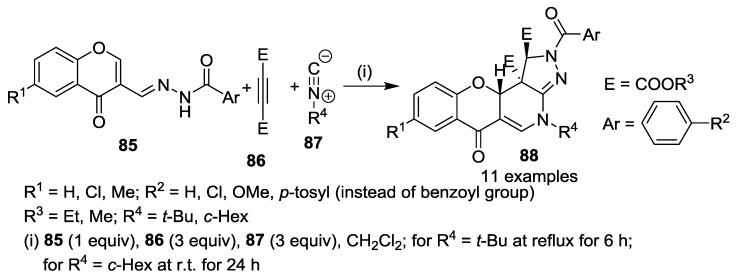
Diastereoselective one-pot synthesis of ABCD-fused chromeno[2,3-*d*]pyrazolo[3,4-*b*]pyridines **88 [56]**.

Novel ABCD-fused chromenopyrazolopyridines **88** were synthesized by a multicomponent reaction of chromone-3-benzoylhydrazones **85** with acetylenedicarboxylates **86** and isocyanides **87** in dichloromethane (Scheme 26). The reaction was diastereoselective affording the tetracyclic benzopyrone derivatives **88**, containing three stereogenic centres, in moderate to good yields (52–65%) [56]. These compounds **88** are related to the alkaloid (+/−)-elaeocarpine having the same three fused-ring core and one derivative was identified as a promising lead compound for the design of novel tetracyclic chromenopyrazolopyridines combining antilipid peroxidation and lipoxygenase inhibitory activities [56].

**Scheme 27:**
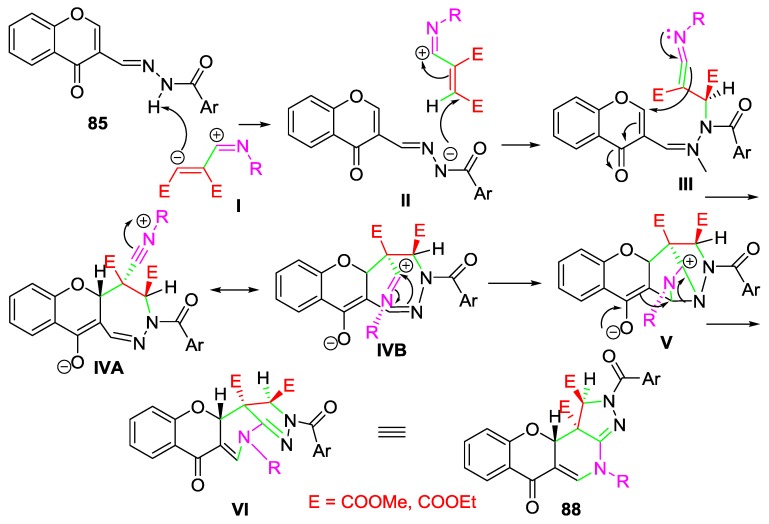
Plausible reaction mechanism for the formation of chromenopyrazolopyridines **88** [56].

A plausible mechanism for the formation of the tetracyclic chromenopyrazolopyridines **88** was proposed (Scheme 27), where the initially formed isocyanide-acetylenedicarboxylate zwitterionic intermediate **I** abstracts preferentially the acidic NH hydrazone proton leading to intermediate **II**. The ester group at position 1 and the isocyanide group at position 2 most probably adopt a favored antiperiplanar conformation, while the two ester groups have the less energy demanding *syn*-conformation. Then, a ring closure occurs leading to intermediate **IVA**, without opening of the pyran ring. After formation of **IVA**, the resonance form **IVB** can be obtained by moving the electron pair on N4 and folding of the diazepine ring. In **IVB** an intramolecular [2+2] stepwise polar cycloaddition of C7=N8 double bond with isocyanide group C3=N4 is possible affording regioselectively a diazetidine intermediate **V**. In the next step, **V** undergoes an electrocyclic ring opening, supported by the adjacent enolate anion, to relieve the extra stretch, bend, torsion and Van der Waals energy and giving the isolated tetracyclic benzopyrone **88** (Scheme 27) [56].

**Scheme 28:**
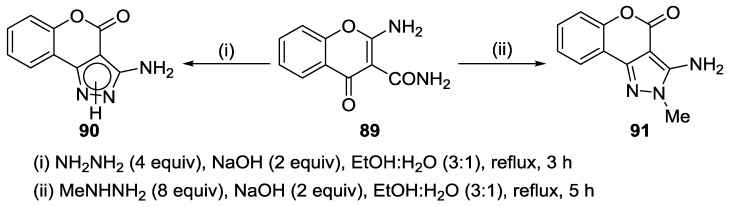
Synthesis of 3-aminochromeno[4,3-*c*]pyrazol-4-ones **90** and **91** from the reaction of 2-amino-3-carbamoylchromone **89** with hydrazines [57].

The reaction of 2-amino-3-carbamoylchromone **89** with hydrazines afforded 3-aminochromeno[4,3-*c*]pyrazol-4-ones **90** and **91** (Scheme 28). The reaction with hydrazine afforded compound **90** in 55% yield, which in DMSO-*d*_6_ was found to exist as a mixture of two tautomers in the ratio 77:23, being 2*H*-tautomer the major one. The reaction with methylhydrazine afforded compound **91** in 35% yield and the structure of the obtained regioisomer was confirmed based on two-dimensional nuclear Overhauser spectroscopy (2D NOESY) experiment, which exhibited a clear cross-peak between the protons of the Me and NH_2_ groups [57]. Compounds **90** and **91**, which are coumarins having a heterocyclic moiety like pyrazole at positions 3 and 4 are key substrates for the preparation of various medicinal drugs [57].

**Scheme 29:**
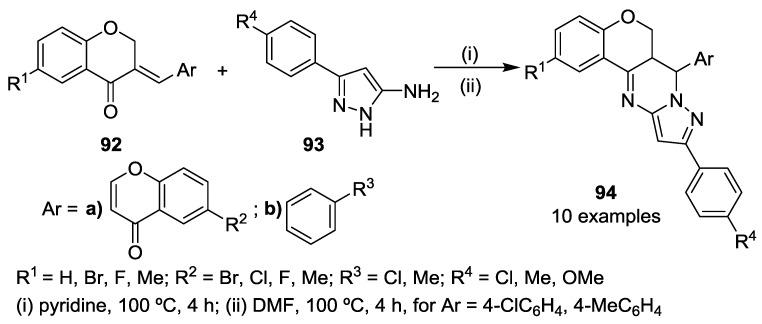
Synthesis of 7-aryl/chromonyl-benzopyrano[4,3-*d*]pyrazolo[l,5-*a*]pyrimidines **94** [58].

The reaction of chromanones **92a** with 5-amino-3-arylpyrazoles **93** in pyridine at 100 °C gave fused 7-chromon-3-ylbenzopyrano[4,3-*d*]pyrazolo[l,5-*a*]pyrimidines **94a** in moderate yields (51–57%) (Scheme 29) [58]. Similarly, the reaction of chromanones **92b** with **93** in DMF gave 7-aryl-benzopyrano[4,3-*d*]pyrazolo[l,5-*a*]pyrimidines **94b** in 48–51% yield (Scheme 29) [58]. The formation of compounds **94a** and **94b** result from the condensation between amino group of 5-aminopyrazole **93** with carbonyl group of **92** followed by Michael addition on the double bond by the nitrogen pyrazole ring [58].

## 4. Chromones as Starting Materials in the Synthesis of 3(5)-(2-Hydroxyaryl)pyrazoles

### 4.1. Reaction with Hydrazine

**Scheme 30:**
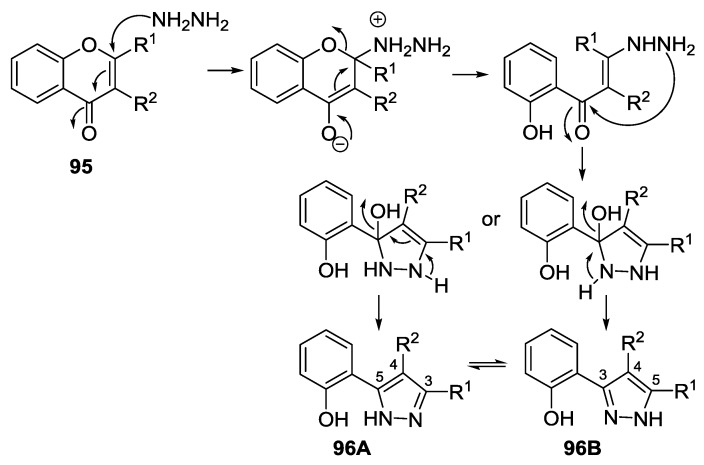
Mechanism for the synthesis of 3(5)-(2-hydroxyaryl)pyrazoles **96** from the reaction of chromones **95** with hydrazine under neutral conditions.

Since 1940s and 1950s is known that 3(5)-(2-hydroxyaryl)pyrazoles **96** (Scheme 30) are the main products obtained from the reaction of chromones with hydrazine hydrate, instead of the hydrazones derived from the starting chromones [59,60,61]. The reaction mechanism involves nucleophilic attack at C-2 of the chromone **95** with consequent ring opening, followed by an intramolecular hydrazone formation (Scheme 30).

**Scheme 31:**
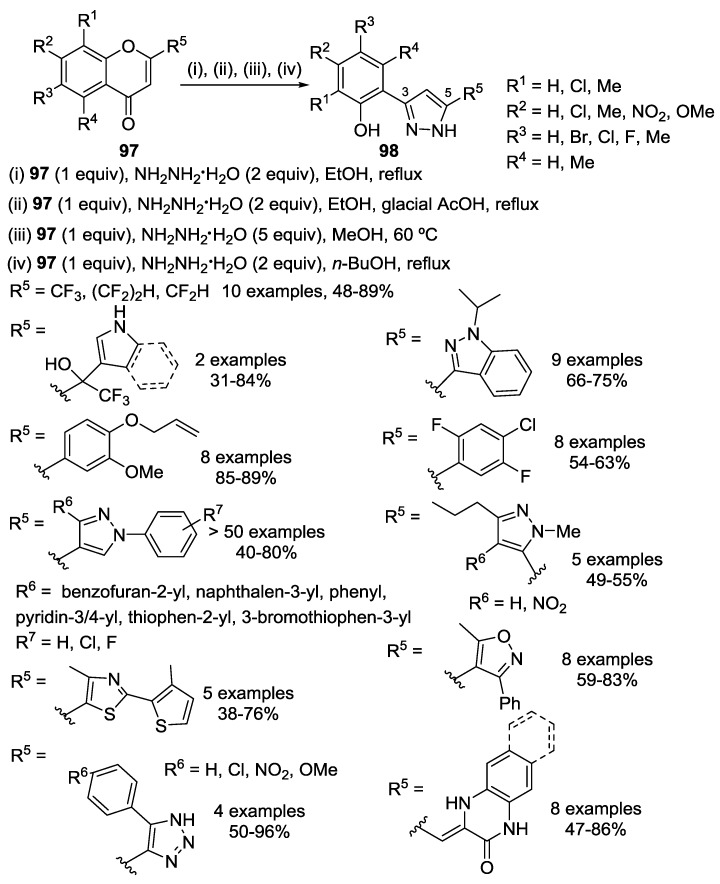
Synthesis of 3(5)-(2-hydroxyaryl)-5(3)-substituted pyrazoles **98** from the reaction of 2-substituted chromones **97** with an excess of hydrazine hydrate, using different reaction conditions [8,18,19,20,21,62,63,64,65,66,67,68,69,70,71,72,73,74]. To simplify the schemes, in all the reactions that can include both 3- and 5-(2-hydroxyaryl)pyrazoles, only the 3-isomer will be represented.

This selective and fast procedure, involving an excess of hydrazine hydrate in refluxing ethanol, was applied to a wide variety of 2-substituted chromones **97** to afford several 3(5)-(2-hydroxyaryl)-5(3)-substituted pyrazoles **98**. The substitution at carbon C-2 of the chromone unit can include polyfluoroalkyl [62,63], 1-isopropyl-1*H*-indazol-3-yl [64], 4-(allyloxy)-3-methoxyphenyl-1-yl [65], 4-chloro-3,4-difluorophenyl-1-yl [66], 1-aryl-3-(benzofuran-2-yl/naphthalen-3-yl/phenyl/pyridin-3/4-yl, thiophen-2-yl/3-bromothiophen-2-yl)pyrazol-4-yl [18,19,20,21,67,68,69,70], 1-methyl-3-propyl-4-substituted pyrazol-5-yl [8], 2-(3-methylthiophen-2-yl)-1,3-thiazol-5-yl [71] substituents (step (i), Scheme 31). Using a 5-methyl-3-phenylisoxazol-4-yl group as C-2 substituent, the reaction occurs in the presence of glacial acetic acid to afford 3(5)-(2-hydroxyaryl)-5(3)-(5-methyl-3-phenylisoxazol-4-yl)-1*H*-pyrazoles, in good yields (59–83%) (step (ii), Scheme 31) [72]. Replacing ethanol by another alcohol as solvent, a series of 2-(5-aryl-1,2,3-triazol-4-yl)chromones in methanol (step (iii), (Scheme 31) or 2-[2-(quinoxalin-2-one-3-yl)methylidene-chromones in *n*-butanol (step (iv), Scheme 31) undergo condensation with hydrazine hydrate to furnish 5(3)-(5-aryl-1,2,3-triazol-4-yl)-3(5)-(2-hydroxyaryl)-1*H*-pyrazoles [73] or 3(5)-(2-hydroxy-aryl)-5(3)-[2-(quinoxalin-2-one-3-yl)methylidene]-1*H*-pyrazoles [74], respectively. Biological evaluation of a wide range of 3(5)-(2-hydroxyaryl)pyrazoles **96** were performed, mainly as antimicrobial and antioxidant agents [8,18,19,20,21,64,65,66,67,70,71,72].

**Scheme 32:**
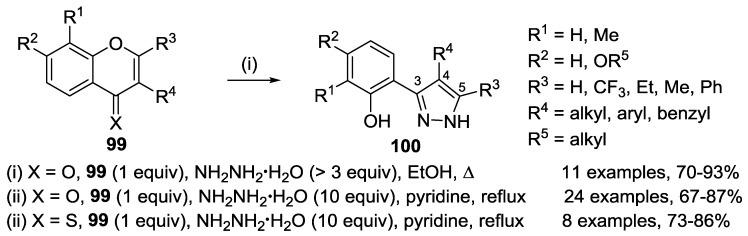
Synthesis of 3(5)-(2-hydroxyaryl)-4,5(3)-disubstituted-pyrazoles **100** from the reaction of 2,3-disubstituted-(thio)chromones **99** with an excess of hydrazine hydrate, using different reaction conditions [77,78,79,80].

Treating 5-[(chromon-3-yl)methylene]-1,3-thiazolidine-2,4-dione with equimolar amount of hydrazine hydrate in refluxing ethanol and in the presence of sodium ethoxide gave the corresponding 3(5)-(2-hydroxyphenyl)-4-[(1,3-thiazolidine-2,4-dione)methylene]pyrazole while using glacial acetic acid, the acetylated derivative 1-acetyl-5-(2-hydroxyphenyl)-4-[(1,3-thiazolidine-2,4-dione)methylene]pyrazole was obtained [75]. Both pyrazole derivatives presented moderate antifungal activity against *Candida albicans* fungi strain [75]. A couple of 3-(2-hydroxyphenyl)-5-{3/4-[3-(2-hydroxyphenyl)pyrazol-5-yl]phenyl}pyrazoles arise from the reaction of 3′/4′-(2-chromonyl)flavones with an excess of hydrazine hydrate in refluxing methanol [76]. Several 3(5)-(2-hydroxyaryl)-4,5(3)-disubstituted pyrazoles **100** were also obtained from the reaction of 2,3-disubstituted (thio)chromones **99** with hydrazine hydrate in refluxing ethanol [77,78] or in hot pyridine (Scheme 32) [79,80]. Substituents at carbon C-2 include alkyl or aryl groups while at carbon C-3 present alkyl, aryl or benzyl groups.

**Scheme 33:**
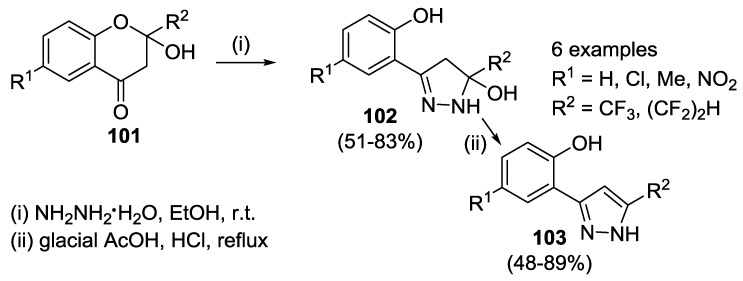
Synthesis of 3(5)-(2-hydroxyaryl)-5(3)-substituted-pyrazoles **103** from the reaction of 2-hydroxy-2-polyfluoroalkylchromanones **101** with an excess of hydrazine hydrate [62].

3(5)-(2-Hydroxyaryl)-5(3)-polyfluoroalkylpyrazole derivatives **103** were also prepared in two steps starting from 2-hydroxy-2-polyfluoroalkylchromanones **101** and hydrazine hydrate to afford 5-hydroxy-3-(2-hydroxyaryl)-5-polyfluoroalkyl-∆^2^-pyrazolines **102**, which undergo subsequent treatment with boiling glacial acetic acid in the presence of HCl (Scheme 33) [62]. A single example of the reaction of 6-methyl-2-trifluoromethylchromene-4-thione with hydrazine hydrate in ethanol at room temperature provided the respective 3(5)-(2-hydroxy-5-methylphenyl)-5(3)-trifluoromethylpyrazole in 65% yield [81]. On the other hand, the reaction of diethyl [(4-chlorophenylamino)(6-methylchromon-3-yl)methyl]phosphonate with hydrazine hydrate and sodium ethoxide in refluxing ethanol led to the 3(5)-(2-hydroxy-5-methylphenyl)-4-(α-aminophosphonate)pyrazole in 97% yield [82]. This pyrazole showed a moderate antimicrobial activity and a good antioxidant effect [82].

**Scheme 34:**
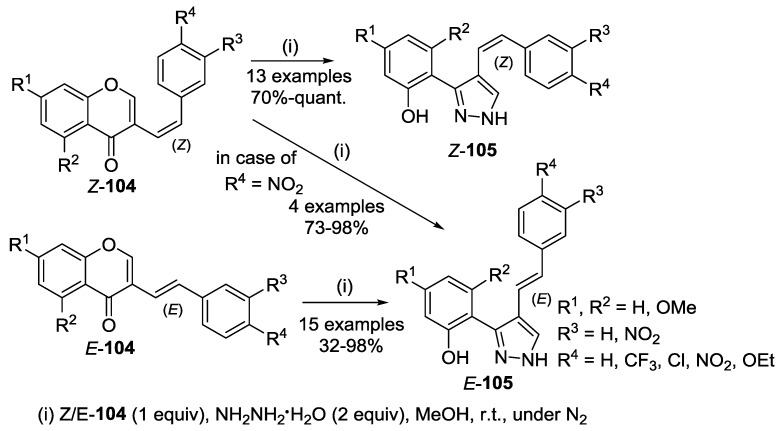
Synthesis of (*Z*)- and (*E*)-3(5)-(2-hydroxyaryl)-4-styrylpyrazoles *Z*-**105** and *E*-**105** from the reaction of (*Z*)- and (*E*)-3-styrylchromones *Z*-**104** and *E*-**104** with hydrazine hydrate [83,84].

**Scheme 35:**
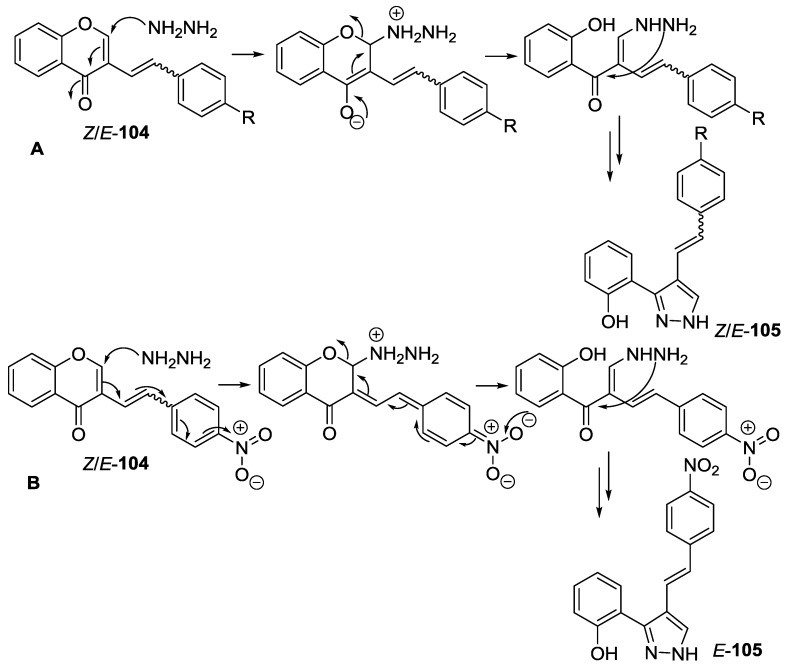
(**A**): Generic mechanism proposed for the synthesis of (*Z*)- and (*E*)-3(5)-(2-hydroxyphenyl)-4-styrylpyrazoles *Z*-**105** and *E*-**105**, starting from, respectively, (*Z*)- and (*E*)-3-styrylchromones *Z*-**104** and *E*-**104** and hydrazine hydrate. (**B**): Mechanism proposed for the synthesis of (*E*)-3(5)-(2-hydroxyphenyl)-4-(4-nitrostyryl)pyrazoles *E*-**105**, starting from (*Z*)- and (*E*)-4′-nitro-3-styrylchromones *Z*-**104** and *E*-**104** and hydrazine hydrate [83,84].

An efficient and selective methodology for the synthesis of (*Z*)- and (*E*)-3(5)-(2-hydroxyaryl)-4-styrylpyrazoles *Z*-**105** and *E*-**105** (Scheme 34) was reported by Silva and coworkers [83,84]. The reaction of (*Z*)- and (*E*)-3-styrylchromones *Z*-**104** and *E*-**104** with an excess of hydrazine hydrate in methanol at room temperature provided the respective (*Z*)- and (*E*)-3(5)-(2-hydroxyaryl)-4-styrylpyrazoles (Scheme 34 and Scheme 35A). However, when chromones present a nitro group substituent at B-ring, both (*Z*)- and (*E*)-isomers, *Z*-**104** and *E*-**104**, afforded only (*E*)-3(5)-(2-hydroxyaryl)-4-(4-nitrostyryl)pyrazoles *E*-**105** (Scheme 34 and Scheme 35B). The mechanism proposed by the authors considers that although both nitro and carbonyl groups are strong electron-withdrawing groups, the primer is the most powerful one. So, after the nucleophilic attack at C-2 of the chromone nucleus, the electronic conjugation should move towards the 4′-nitro-3-styryl moiety instead of the 4-carbonyl group (Scheme 35B).

This conjugate addition allowed the (*Z*)→(*E*) isomerisation of the vinylic double bond of the styryl group, to the most stable configuration, and consequent ring opening. The last step of this reaction involves pyrazole ring closure by an intramolecular hydrazone formation (Scheme 35B).

Condensation reactions of hydrazine hydrate with 3-[bis(diaryl)methyl]chromones in refluxing ethanol gave 4-[bis(diaryl)methyl]-3(5)-(2-hydroxyphenyl)pyrazoles [85]. Similarly, 3-[bis(indol-3-yl)methyl]chromones in refluxing isopropanol provided 3(5)-(2-hydroxyphenyl)-4-[bis(indol-3-yl)methyl]pyrazoles [86].

**Scheme 36:**
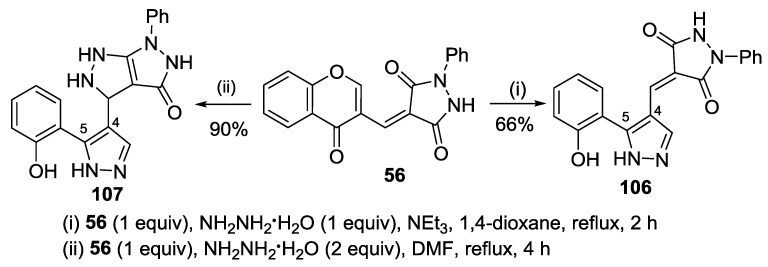
Reaction of 4-(chromenylmethylene)pyrazolidinedione **56** with hydrazine hydrate carried out in the presence of different solvents [47].

Regioselective condensation reactions of compounds containing a pyrone and an exocyclic enone reactive system, with hydrazine hydrate in different reaction conditions were disclosed. Thus, the reaction of 4-(chromenylmethylene)pyrazolidinedione **56** with equimolar amount of hydrazine hydrate in the presence of triethylamine in 1,4-dioxane led to pyrone ring opening to provide the corresponding 4-{[5-(2-hydroxyphenyl)-1*H*-pyrazol-4-yl]methylene}-1-phenyl-pyrazolidine-3,5-dione **106** while in a 1:2 ratio in DMF, both pyrone and enone systems are reactive giving rise to 4-[5-(2-hydroxyphenyl)-1*H*-pyrazol-4-yl]-1-phenyl-1,5,6,6a-tetrahydropyrazolo[3,4-*c*]pyrazol-3(2*H*)-one **107**, in 90% yield (Scheme 36) [47].

**Scheme 37:**
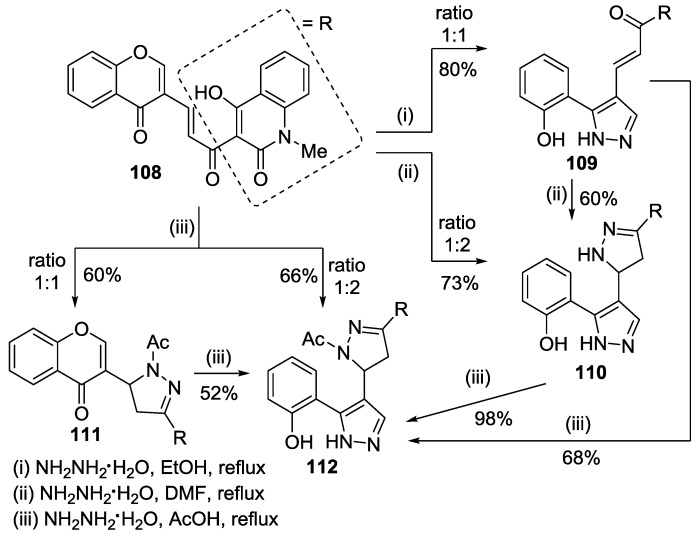
Reaction of 4-hydroxy-1-methyl-3-[(*E*)-3-(chromen-3-yl)acryloyl]quinolin-2(1*H*)-one **108** with hydrazine hydrate carried out in the presence of different solvents [87].

Abass and coworkers reported the condensation reaction of 4-hydroxy-1-methyl-3-[(*E*)-3-chromon-3-yl)acryloyl]quinolin-2(1*H*)-one **108** with an equimolar amount of hydrazine in boiling ethanol to afford pyrazoles **109** while using 2 equivalent (equiv) of hydrazine in refluxing DMF led to the pyrazole-pyrazoline derivative **110** [87]. When the reaction was performed in boiling acetic acid, with an equimolar amount of hydrazine, the pyrone ring maintains intact delivering the *N*-acetylated pyrazoline derivative **111**; with 2 equiv of hydrazine, *N*-acetylated pyrazoline-pyrazole derivative **112** was isolated. Moreover, derivative **112** can also be synthesized from the compounds **109**, **110** and **111** with hydrazine hydrate in boiling DMF (Scheme 37) [87].

**Scheme 38:**
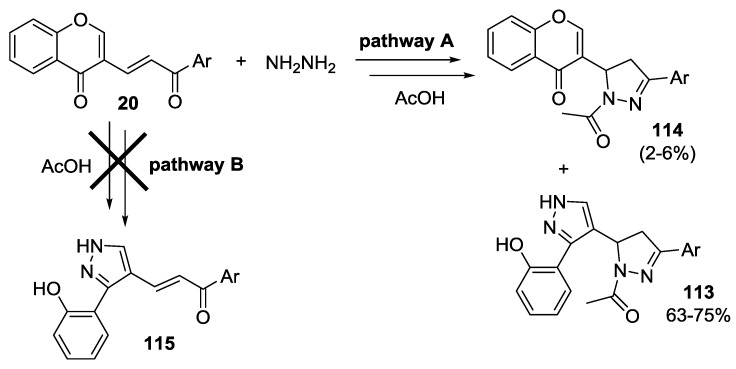
Pathways for the reaction of 3-(3-aryl-3-oxoprop-1-en-1-yl)chromones **20** with an excess of hydrazine hydrate in hot acetic acid [88,89].

1-Acetyl-3-aryl-5-[3-(2-hydroxyphenyl)pyrazol-4-yl]-2-pyrazoline derivatives **113** were obtained as major products (63–75% yield) from the reaction of 3-(3-aryl-3-oxoprop-1-en-1-yl)chromones **20** with an excess of hydrazine hydrate in hot acetic acid (Scheme 38). From this reaction, 1-acetyl-3-aryl-5-(3-chromonyl)-2-pyrazolines **114** were also obtained as by products (2–6% yield) [88]. The authors pointed two possible pathways for the formation of pyrazolyl-2-pyrazolines: pathway A involving the formation of the 1-acetyl-3-aryl-5-(3-chromonyl)-2-pyrazolines **114** in the first step, as in standard reactions between α,β-unsaturated ketones and hydrazines [89] or pathway B through the reaction of 3-chromonyl group and hydrazine before the reaction of α,β-unsaturated ketone moiety affording the 3,4-disubstituted pyrazoles **115**. Both intermediates **114** and **115** can then react with another equivalent of hydrazine to provide the pyrazolyl-2-pyrazolines **113** (Scheme 38). So, the experimental results supported that this reaction must proceed through the first possibility, since 1-acetyl-3-aryl-5-(3-chromonyl)-2-pyrazolines **114** were isolated as minor reaction products [88].

**Scheme 39:**
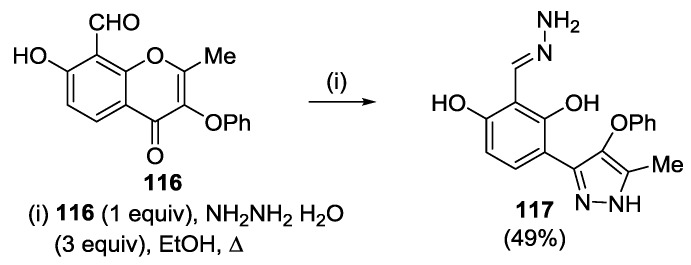
Reaction of 8-formyl-7-hydroxy-2-methyl-3-phenoxychromone **116** with an excess of hydrazine hydrate [90].

The excess of hydrazine hydrate used in the reaction of 8-formyl-7-hydroxy-2-methyl-3-phenoxychromone **116** in refluxing ethanol led to the isolation of a unique structure **117** (Scheme 39) containing a hydrazone moiety, formed from the reaction of hydrazine with the 8-formyl group, but also the pyrazole ring, resulted from the reaction of hydrazine and recyclization of the γ-pyrone ring of the starting chromone [90].

**Scheme 40:**
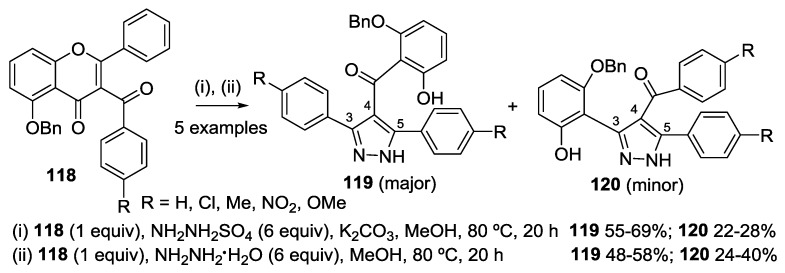
Reaction of 3-aroyl-2-aryl-5-benzyloxychromones **118** with different sources of hydrazine [91].

The presence of two carbonyl groups in the 3-aroyl-2-aryl-5-benzyloxychromone structures **118** allowed the formation of two different types of pyrazoles when the reaction occurred in the presence of hydrazine, generated in situ by addition of potassium carbonate to hydrazinium sulfate or from the commercially available hydrate, in methanol at 80 °C (Scheme 40) [91]. Better overall yields were obtained with hydrazine hydrate, being in all cases the 3,5-diaryl-4-(2-benzyloxy-6-hydroxybenzoyl)pyrazoles **119** isolated as major products along with 4-aroyl-5-aryl-3-(2-benzyloxy-6-hydroxyphenyl)pyrazoles **120** as minor compounds. These results pointed that the carbonyl group of 3-aroyl group is more reactive than the chromone carbonyl group [91].

**Scheme 41:**
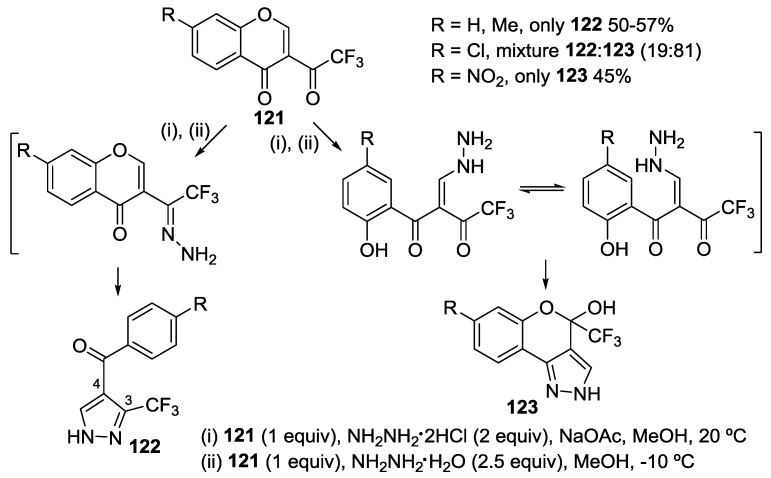
Reaction of 3-(polyfluoroacyl)chromones **121** with different sources of hydrazine [92].

**Scheme 42:**
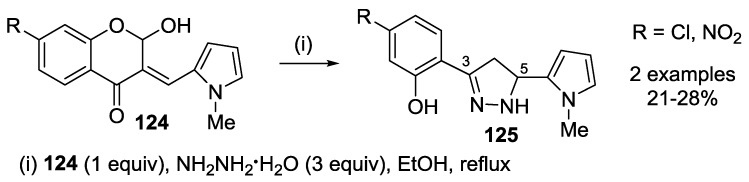
Synthesis of 3-(2-hydroxyphenyl)-5-(1-methylpyrrol-3-yl)-2-pyrazolines **125** from the reaction of (*E*)-2-hydroxy-3-(1-methylpyrrol-2-ylmethylene)chromanones **124** with hydrazine hydrate in refluxing ethanol [86].

The two carbonyl groups of 3-(polyfluoroacyl)chromones **121** were also reactive with different sources of hydrazine in different reaction conditions [92]. Thus, using hydrazine dihydrochloride in the presence of anhydrous sodium acetate in methanol at room temperature yielded different products according to the nature of the substituents in the chromone ring. Chromones **121** (R = H, Me) afforded only 4-(2-hydroxylbenzoyl)-3-(trifluoromethyl)pyrazoles **122** whereas chromone **121** (R = NO_2_) gave solely 4-(trifluoromethyl)-2,4-dihydrochromeno[4,3-*c*]pyrazol-4-ol **123** (Scheme 41), formed through nucleophilic 1,4-addition with subsequent pyrone ring opening and heterocyclization reactions. Chromone **121** (R = Cl) prompted a mixture of compounds **122**:**123**, in a ratio of 1:4. On the other hand, the reaction of the 3-(polyfluoroacyl)chromones **121** with an aqueous solution of hydrazine hydrate in methanol at −10 °C afforded the same derivatives in the case of chromones substituted with chloro and nitro groups while for the unsubstituted chromone and substituted with a methyl group, starting chromones underwent detrifluoroacetylation and deformylation to afford the corresponding 2′-hydroxyacetophenones [92]. A couple of 3-(2-hydroxyphenyl)-5-(1-methylpyrrol-3-yl)-2-pyrazolines **125** were obtained from the reaction of (*E*)-2-hydroxy-3-(1-methylpyrrol-2-ylmethylene)chromanones **124** with 3 equiv of hydrazine hydrate in refluxing ethanol (Scheme 42). The mechanism proposed involves deformylation of the starting chromanones to the corresponding chalcones followed by heterocyclization reactions [86].

### 4.2. Reaction with Methylhydrazine

**Scheme 43:**
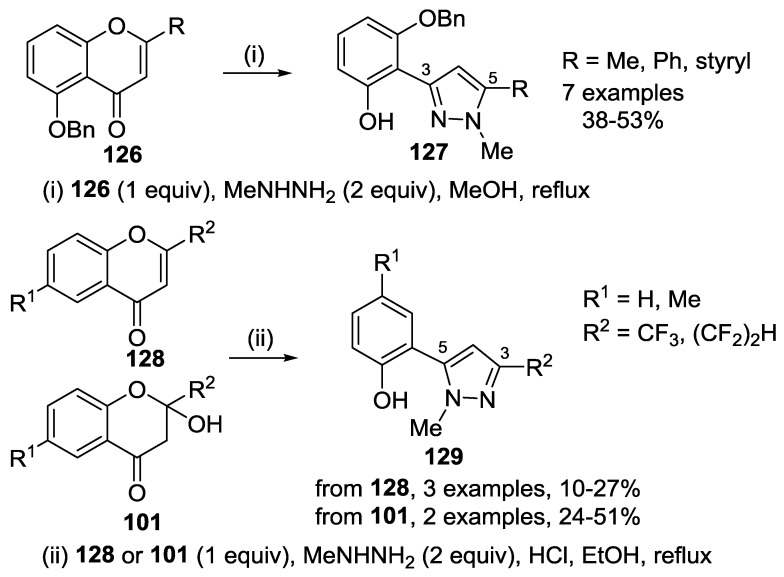
Different pyrazole isomers **127** and **129** were obtained from the reaction of compounds **126**, **128** and **101** with methylhydrazine in neutral and in acidic medium [62,93].

**Scheme 44:**
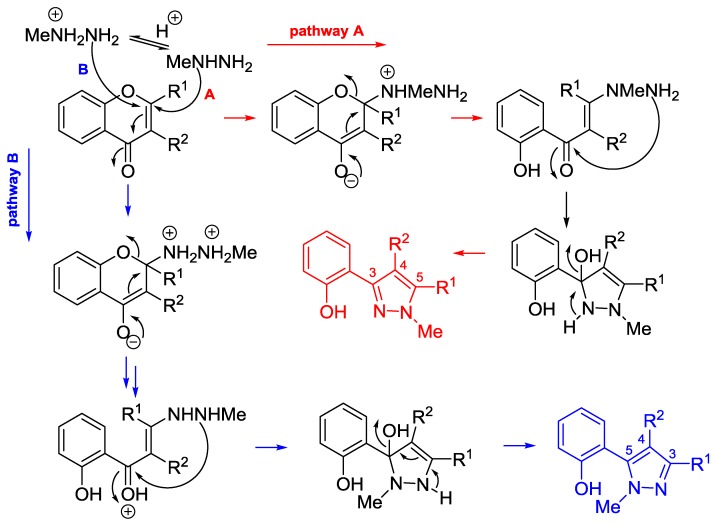
Mechanism of the reaction of chromones with methylhydrazine in neutral (pathway A) and in acidic (pathway B) medium.

In 2000, Silva and coworkers reported the reaction of 5-benzyloxy-2-(methyl/phenyl/ styryl)chromones **126** with four batches (2 equiv each) of methylhydrazine in refluxing methanol for 24 h. From this reaction only 3-(2-benzyloxy-6-hydroxyphenyl)-1-methylpyrazoles **127** were obtained in 38–53% yield, recovering a significant amount of the starting chromones **126** (33–50%) (Scheme 43) [93]. From these results it was concluded that the secondary amino group of the methylhydrazine attacks carbon C-2 of the chromone moiety with consequent ring opening (Scheme 44, pathway A), followed by intramolecular hydrazone formation to afford solely the corresponding 3-(2-hydroxyphenyl)-1-methylpyrazoles. Unlike described before, when 2-polyfluoroalkylchromones **128** or 2-hydroxy-2-polyfluoroalkylchroman-4-ones **101** reacted with methylhydrazine in boiling ethanol in the presence of HCl a series of 5-(2-hydroxyaryl)-1-methyl-3-polyfluoroalkylpyrazoles **129** were obtained regioselectively (Scheme 43) [62]. In this reaction performed in acidic medium (Scheme 44, pathway B), the protonation of the NHMe group occurs and consequently it is the nucleophile attack of the primary amino group of methylhydrazine to the carbon C-2 of the chromone unit that starts the sequence.

**Scheme 45:**
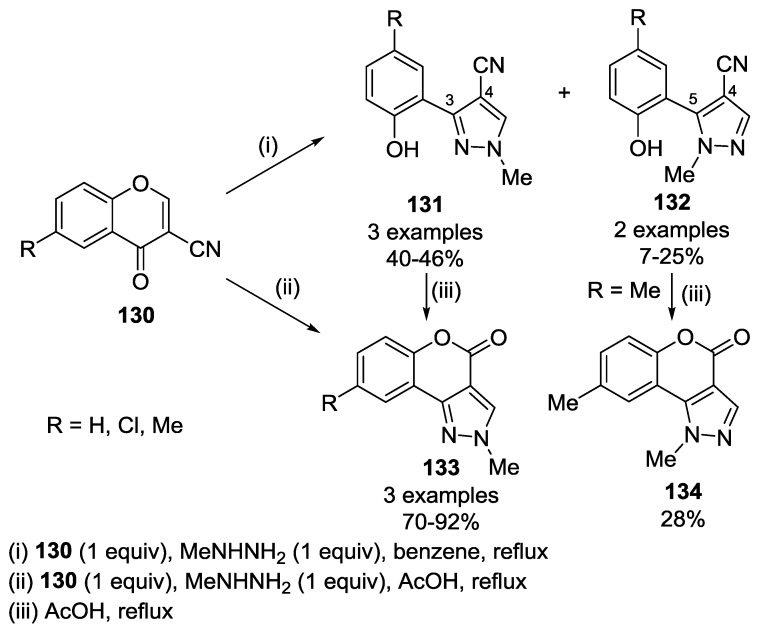
Reaction of 3-cyanochromones **130** with methylhydrazine carried out in different solvents [94].

On the other hand, a mixture of 4-[bis(4-diethylaminophenyl)methyl]-3(5)-(2-hydroxyphenyl)-1-methyl-1*H*-pyrazoles were achieved from the reaction of 3-[bis(4-diethylaminophenyl)-methyl]chromone with an excess of methylhydrazine in refluxing ethanol [85]. Varying the conditions in the reaction of 3-(polyfluoroacyl)chromones with methylhydrazine, 4-(2-hydroxybenzoyl)-1-methyl-3-(trifluoromethyl)pyrazoles and/or 2-methyl-4-(trifluoromethyl)-2,4-dihydrochromeno[4,3-*c*]pyrazol-4-ols were obtained as main products [92]. Other regioselective reactions were achieved using equimolar mixtures of 3-cyanochromones **130** and methylhydrazine, varying the solvent [94]. In refluxing benzene, 4-cyano-3-(2-hydroxyaryl)-1-methylpyrazoles **131** were obtained as major products (40–46%) while the isomeric 5-arylpyrazoles **132** were obtained in smaller amounts; under boiling acetic acid, only 2-methylchromeno[4,3-*c*]pyrazol-4(2*H*)-ones **133** were isolated in 70–92% yields (Scheme 45). To note that these arylpyrazoles **131** and **132** can be cyclized in boiling acetic acid into the respective chromenopyrazolones **133** and **134**, with better yields in the two-steps approach [94].

**Scheme 46:**
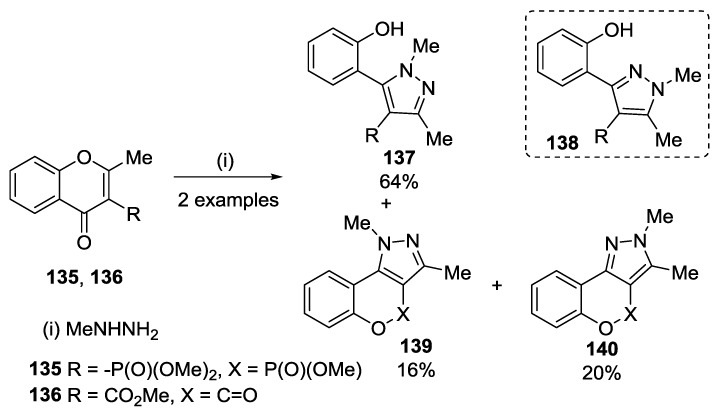
Reaction of phosphonic chromone **135** and its C-3 methoxycarbonyl analogue **136** with methylhydrazine, under solvent-free conditions [95].

Interestingly, in 2004 Budzisz and coworkers reported the isolation of similar products of those described before, from the reaction of phosphonic chromone **135** and its C-3 methoxycarbonyl analogue **136** with an equimolar amount of methylhydrazine at room temperature, under solvent-free conditions. In this case, 3-(2-hydroxyphenyl)pyrazoles **138** were not isolated from the reaction mixture being 5-(2-hydroxyphenyl)-3-methyl-4-phosphonyl pyrazoles **137** obtained as major compounds along with tricyclic compounds **139** and **140**, as minor products. The formation of compounds **139** and **140** result from the intramolecular transesterification of the formed pyrazoles **137** and **138**, respectively (Scheme 46) [95]. The addition of a second equiv of methylhydrazine to the reaction mixture improved the yield of the tricyclic compounds [96].

A couple of *N*-methylated pyrazolyl-2-pyrazoline derivatives arise in moderate yield from the reaction of (*E*)-3-[3-(2-hydroxyaryl)-3-oxoprop-1-en-1-yl]chromones with two equiv of methyl-hydrazine in tetrahydrofuran (THF) at room temperature. The mechanism involves a domino sequence of nucleophilic attack at the chromone C-2 with pyrone ring-opening and pyrazole ring closure, together with a 1,4-aza-Michael addition to the exocyclic enone system followed by the formation of the 2-pyrazoline ring [97].

### 4.3. Reaction with Phenylhydrazine

**Scheme 47:**
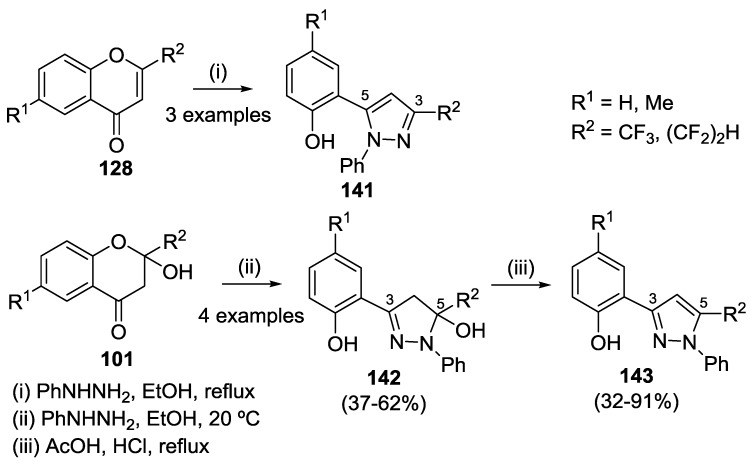
Regioselective reaction of chromones **128** and chromanonols **101** with phenylhydrazine carried out at different temperatures [62].

**Scheme 48:**
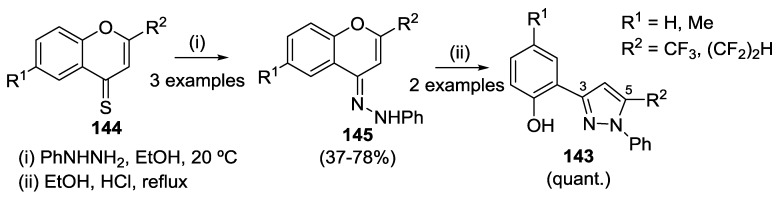
Regioselective reaction of chromene-4-thiones **144** with phenylhydrazine and subsequent acidic ring-closure [81].

Sosnovskikh and coworkers reported in 2002 the reaction of phenylhydrazine with 2-poly-fluoroalkylchromones **128** in refluxing ethanol and the corresponding 5-(2-hydroxyaryl)-1-phenyl-3-polyfluoroalkylpyrazoles **141** were isolated in poor yields (13–20%) (Scheme 47) [62]. Alternatively, reacting 2-hydroxy-2-polyfluoroalkylchromanones **101** with phenylhydrazine at room temperature yielded the corresponding 5-hydroxy-3-(2-hydroxyaryl)-1-phenyl-5-polyfluoroalkyl-∆^2^-pyrazolines **142** (37–62% yield), which were converted into the respective 3-(2-hydroxyaryl)-1-phenyl-5-polyfluoroalkylpyrazoles **143** (32–91% yield), after treatment with boiling glacial acetic acid in the presence of HCl [62]. A couple of these 1-Ph-5-R^F^-pyrazoles **143** can also arise from a two-step reaction of 2-polyfluoroalkylchromene-4-thiones **144** with phenylhydrazine for 15 min, in solvent-free conditions, to give chromone *N*-phenylhydrazones **145**, which suffer subsequent acidification with HCl in refluxing ethanol (Scheme 48) [81].

**Scheme 49:**
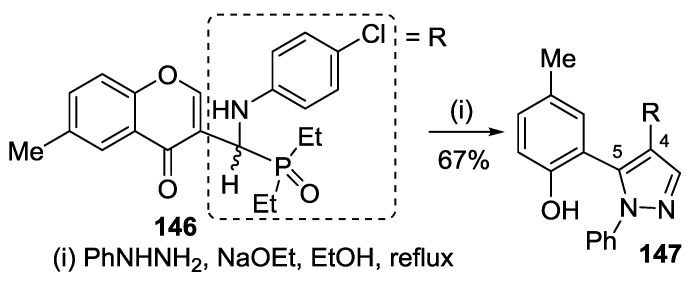
Reaction of diethyl [(4-chlorophenylamino)(6-methylchromon-3-yl)methyl]phosphonate **146** with phenylhydrazine and sodium ethoxide in refluxing ethanol [82].

Lévai et al. studied the reactivity of few 3-arylchromones with phenylhydrazine in hot pyridine. Only those chromones non-substituted at C-2 were allowed to react and a couple of 4-aryl-3(5)-(2-hydroxyaryl)-1-phenylpyrazoles were synthesized [80]. A single example of 1-phenyl-5-(2-hydroxy-5-methylphenyl)-3-(α-aminophosphonate)pyrazole **147** arose in 67% yield from the reaction of diethyl [(4-chlorophenylamino)(6-methylchromon-3-yl)methyl]phosphonate **146** with phenyl-hydrazine and sodium ethoxide in refluxing ethanol (Scheme 49) [82]. Using the same procedure, 5-(2-hydroxyphenyl)-1-phenyl-4-[(1,3-thiazolidine-2,4-dione)methylene]-pyrazole arose from 5-[(chromon-3-yl)methylene]-1,3-thiazolidine-2,4-dione, in 63% yield [75].

**Scheme 50:**
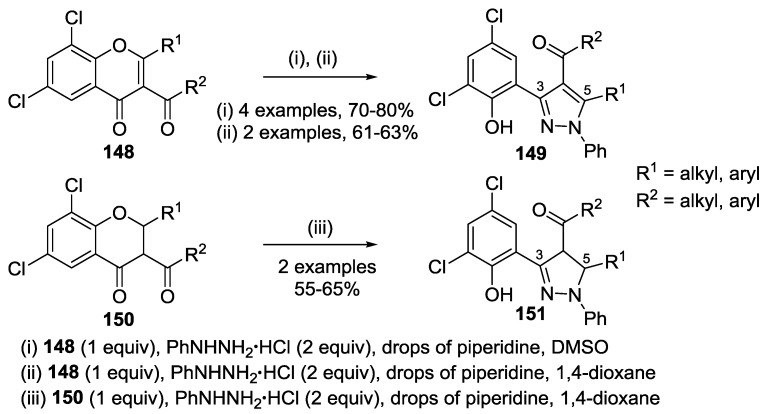
Reaction of 3-aroyl-6,8-dichlorochromones **148** and chromanones **150** with phenylhydrazine hydrochloride carried out in the presence of piperidine, in different solvents [98,99].

**Scheme 51:**
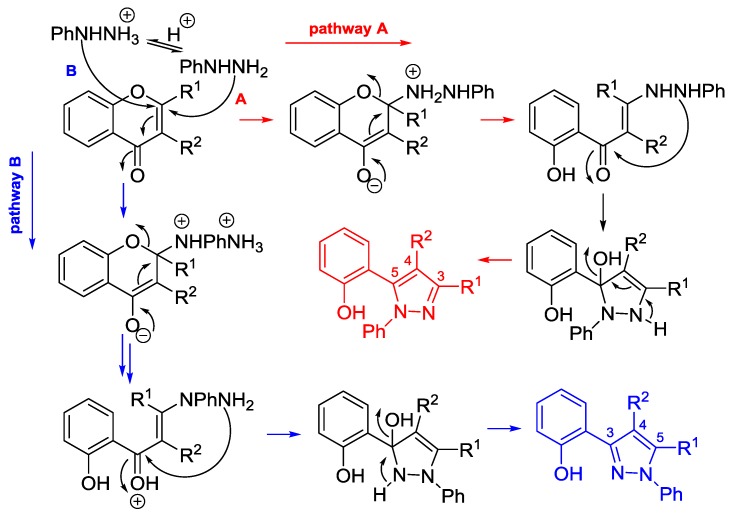
Mechanism of the reaction of chromones with phenylhydrazine in neutral (pathway A) and in acidic (pathway B) medium.

Treating 3-aroyl-6,8-dichlorochromones **148** with phenylhydrazine hydrochloride in the presence of a small amount of piperidine and using DMSO [98] or 1,4-dioxane [99] as solvent furnished the corresponding 4-aroyl-3-(3,5-dichloro-2-hydroxyphenyl)-1-phenylpyrazoles **149** in 61–80% yield (Scheme 50). In addition, replacing chromones **148** by chromanones **150**, 4-aroyl-3-(3,5-dichloro-2-hydroxyphenyl)-1-phenyl-2-pyrazolines **151** were obtained (Scheme 50) [99]. Moderate to good antibacterial activity against *Escherichia coli*, *Staphylococcus aureus*, *Pseudomonas vulgaris* and *Bacillus subtilis* was observed for pyrazoles **149** [98] while the growth promoting effect on some flowering plants were evaluated not only for pyrazoles **149**, but also for 2-pyrazolines **151** [99]. Unlike methylhydrazine, the reactivity of chromones with phenylhydrazine can proceed in two different ways, due to the equilibrium between the non-protonated and protonated form of the more nucleophilic phenylhydrazine amino group (Scheme 51). In neutral conditions (pathway A), the more nucleophilic amino group (NH_2_) attacks the chromone C-2 carbon, in a conjugate-type addition, with consequent pyran ring opening. Then, the intramolecular reaction between the other amino group (NHPh) and the carbonyl unit lead to the 3-(2-hydroxyphenyl)-1-phenylpyrazoles. In acidic medium (pathway B), protonated hydrazine molecule NHPh becomes the nucleophile attacking the chromone C-2 carbon with pyran ring opening. The 5-(2-hydroxyphenyl)-1-phenylpyrazoles arise from intramolecular reaction between the other amino group (NH_2_) and the carbonyl unit (Scheme 51).

**Scheme 52:**
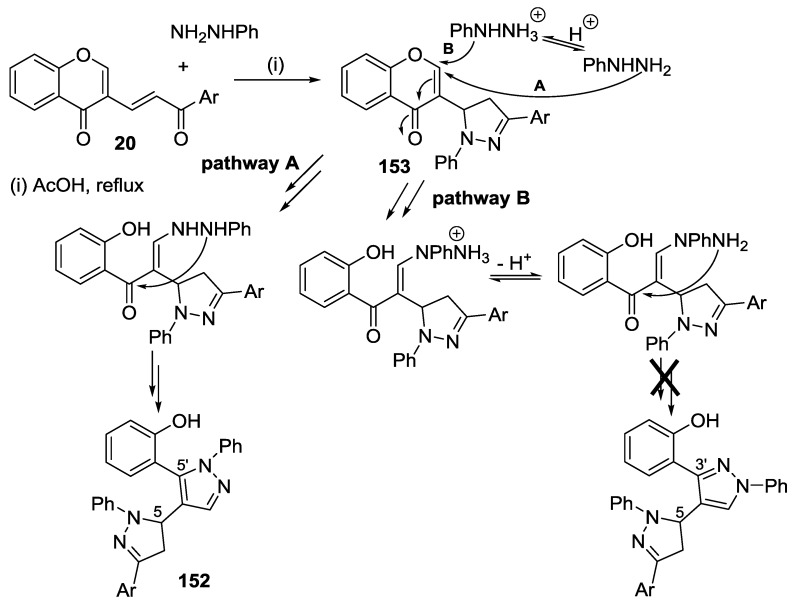
Mechanisms proposed for the reaction of (*E*)-3-[3-(2-hydroxyaryl)-3-oxoprop-1-en-1-yl]chromones **20** with an excess of phenylhydrazine in refluxing acetic acid [100].

An example of the regioselectivity obtained from the reaction of chromones with phenylhydrazine in acidic medium was given by Lévai et al. [100]. Treating (*E*)-3-[3-(2-hydroxyaryl)-3-oxoprop-1-en-1-yl]chromones **20** with an excess of phenylhydrazine in refluxing acetic acid gave only *N*-phenyl pyrazolyl-2-pyrazoline derivatives **152**, in moderate yields (Scheme 52).

According to the results, the reaction starts by the attack of phenylhydrazine to the exocyclic enone to deliver (3-chromonyl)-2-pyrazoline-type compounds **153** as primary reaction intermediates. Then, the reaction in acidic medium proceeds by only one pathway, involving the attack of the more nucleophilic amino group to the chromone C-2 carbon with consequent pyran ring opening and subsequent intramolecular reaction between the other amino group (NHPh) and the carbonyl unit leading to final pyrazolyl-2-pyrazoline derivatives **152** (pathway A, Scheme 52) [100].

### 4.4. Reaction with Other Hydrazines

**Scheme 53:**
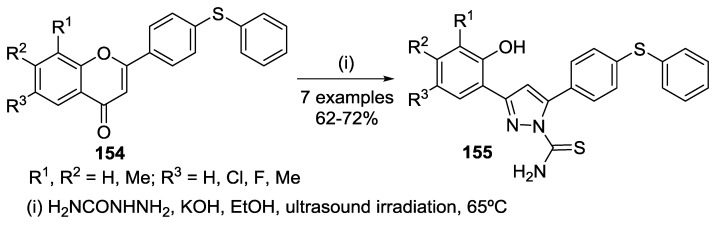
Reaction of 2-[4-(phenylthio)phenyl]chromones **154** with semicarbazide in ethanol and potassium hydroxide, under ultrasound irradiation [102].

Not only simple hydrazines are used to synthesize pyrazole derivatives. The reaction of 3-hydrazino-6-(2-hydroxyphenyl)pyridazine with 2-trifluoromethylchromene-4-thione in refluxing methanol provided the respective 6-(2-hydroxyphenyl)-3-[2-(hydroxyphenyl)-5-trifluoromethyl-1*H*-pyrazol-3-yl]pyridazine in 28% yield. Using the similar chromone derivative, the reaction did not occur [81]. Nucleophiles such as isonicotinic acid, semicarbazide and thiosemicarbazide hydrochloride were allowed to react with 3-benzoyl-6-chloro-2-methylchromone in refluxing methanol giving rise to the corresponding 4-aroyl-5-(5-chloro-2-hydroxyphenyl)-1-isonicotinoyl/carboxamido/thiocarboxamido-3-methylpyrazoles [101]. Other 1-carbothioamide pyrazole derivatives **155** have been accomplished through ring opening of 2-[4-(phenylthio)phenyl]chromones **154** with semicarbazide in ethanol and potassium hydroxide, under ultrasound irradiation (Scheme 53) [102]. Some of the synthesized compounds **155** showed significant antibacterial activity against *Escherichia coli* and *Staphylococcus aureus* and also against the fungal strains *Candida albicans* and *Aspergillus fumigates* [102].

## 5. Miscellaneous

The reactivity of 3-formylchromone derivatives with a series of hydrazines has been studied in detail over the most recent years (for recent reviews see [103,104]). Thus, treating 3-formyl-6-hydroxychromone with an equimolar amount of hydrazine hydrate and phenylhydrazine in refluxing ethanol provided the corresponding 4-(2,5-dihydroxybenzoyl)pyrazoles [46]. Other 4-(2-hydroxyaroyl)pyrazoles were synthesized through the reaction of 3-formylchromone with arylhydrazines in an alcoholic potassium hydroxide solution, under microwave irradiation at 120 °C [105]. A similar one-pot protocol was achieved with the reaction of the parent 3-formylchromone with cyanoacetic acid hydrazide in the presence of sodium ethoxide in refluxing ethanol to afford 4-(2-hydroxybenzoyl)pyrazole [106]. Rindhe and coworkers used a two-step strategy involving the reaction of 3-formylchromones with 2,4-difluorohydrazine using a catalytic amount of acetic acid in ethanol at 40 °C to give the corresponding hydrazones, which after treatment with potassium hydroxide at 50 °C provided the respective 4-(2,5-dihydroxybenzoyl)pyrazoles [107]. From eight of these pyrazoles, one presented a broad spectrum of antibacterial activity against the four tested strains (*Escherichia coli*, *Staphylococcus aureus*, *Pseudomonas aeruginosa* and *Bacillus subtilis*). Moreover, only one of the eight tested compounds showed antifungal activity against *Candida albicans* [107].

**Scheme 54:**
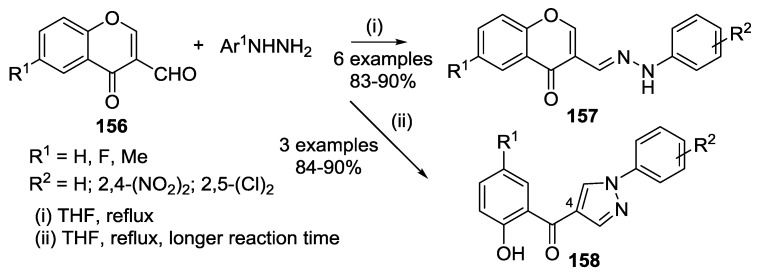
Reaction of 6-substituted 3-formylchromones **156** with aromatic primary hydrazines in refluxing THF [108,109]. Abbreviation: THF, tetrahydrofuran.

The condensation of 6-substituted 3-formylchromones **156** with equimolar amounts of aromatic primary hydrazines in refluxing THF occurs through 1,2-addition reaction at the formyl group to afford the respective hydrazones **157** (Scheme 54). On the other hand, using prolonged heating, the reaction evolved to the formation of 1-aryl-4-(2-hydroxybenzoyl)pyrazoles **158** (Scheme 54), via 1,4-addition reaction with pyrone ring-opening and subsequent recyclization and proton transfer mechanism [108,109]. Both compounds **157** and **158** were screened for their cytotoxic effect against brine shrimps (*Artemia salina*), presenting IC_50_ values of 83–262 μM, considerably higher than the positive control podophyllotoxin (IC_50_ = 5.8 μM). Moreover, the presence of the aromatic fluorine enhances the overall activity when compared with the similar non-fluorinated compounds [108]. In another study, the same compounds **157** and **158** were tested for their antiparasitic activity against promastigotes of *Leishmania mexicana* (Bel 21) and epimastigotes of *Trypanosoma cruzi* (DM28). The IC_50_ values found were 6–53 μM for *L. mexicana* and 4–174 μM for *T. cruzi,* higher than the positive control miltefosine (IC_50_ values of 4.7 μM for *L. mexicana* and 2.3 μM for *T. cruzi*). The most promising compound against both strains was derivative **157** (R^1^ = H, R^2^ = 2,4-(NO_2_)_2_), non-substituted on the chromone unit and bearing a 2,4-dinitrophenyl moiety linked to the hydrazone [109].

A wide range of 1-[4-(4-halophenyl)thiazol-2-yl]-4-(2-hydroxybenzoyl)pyrazoles have been achieved through the reaction of polysubstituted 3-formylchromones with 1-[4-(4-halophenyl)thiazol-2-yl]hydrazines in the presence of potassium hydroxide in refluxing ethanol [23,110].

**Scheme 55:**
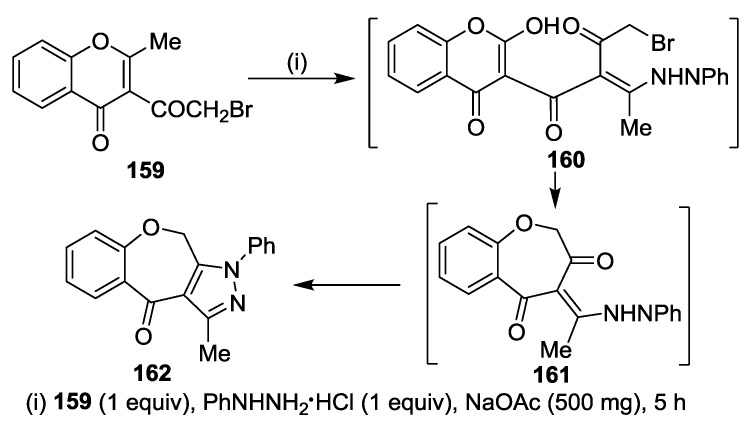
Synthesis of a fused pyrazole **162** by 1,4-addition of phenylhydrazine to 3-bromoacetyl-2-methylchromone **159** [111].

**Scheme 56:**
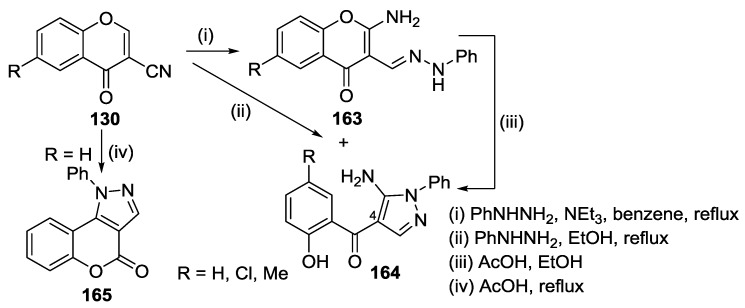
Reaction of 3-cyanochromones **130** with phenylhydrazine, in different reaction conditions [94].

Gosh and coworkers described the 1,4-addition of phenylhydrazine to the α,β-unsaturated carbonyl functionality of 3-bromoacetyl-2-methylchromone **159** with concomitant opening of the pyran ring and formation of intermediate **160** giving rise to the fused pyrazole **162** via **161** (Scheme 55) [111]. In turn, the reaction of 3-cyanochromones **130** with equimolar amounts of phenylhydrazine is solvent-dependent [94]. Thus, in refluxing benzene in the presence of triethylamine, 2-aminochromone *N*-phenylhydrazones **163** were obtained as single products while in refluxing ethanol was isolated a mixture of *N*-phenylhydrazones **163** and 5-amino-4-(2-hydroxyaroyl)-1-phenylpyrazoles **164**, mixture that can be treated with sulfuric acid in ethanol to give solely 5-amino-4-(2-hydroxyaroyl)-1-phenylpyrazoles. In refluxing acetic acid, parent 3-cyanochromone gave the corresponding 1-phenylchromeno[4,3-*c*]pyrazol-4(1*H*)-one **165** in 55% yield (Scheme 56) [94].

**Scheme 57:**
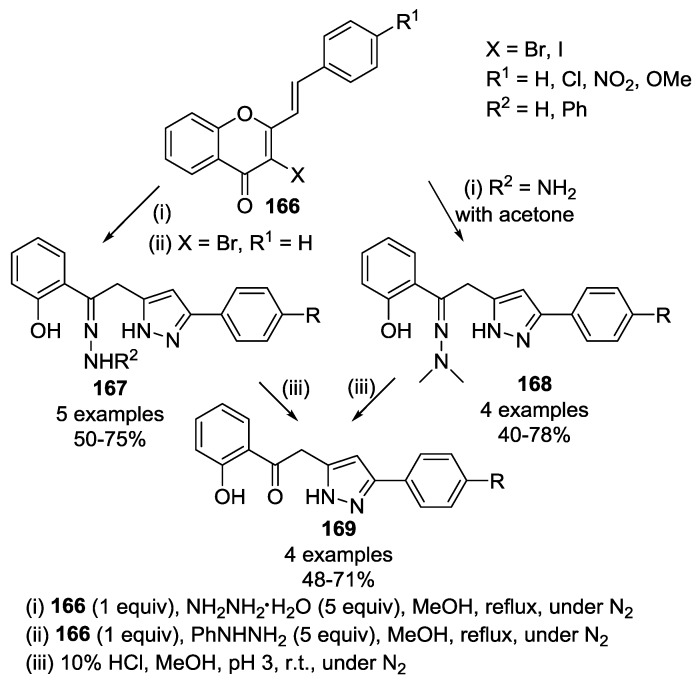
Reaction of (*E*)-3-halo-2-styrylchromones **166** with hydrazine hydrate and phenylhydrazine in refluxing methanol [112].

Treating (*E*)-3-halo-2-styrylchromones **166** with an excess of hydrazine hydrate in refluxing methanol prompted hydrazonepyrazoles **167**, which in acidic conditions suffer hydrazone moiety cleavage to give the respective pyrazoles **169** (Scheme 57) [112]. The most plausible mechanism presented by the authors involves 1,6-conjugate addition of hydrazine to the C-β of chromone **166** with subsequent ring-opening, intramolecular 1,4-conjugate addition to form the intermediate pyrazolines, dehalogenation and finally a 1,5-proton shift process to afford pyrazoles **169**. A similar strategy was applied to the parent (*E*)-3-bromo-2-styrylchromones **166** (R^1^ = H) with phenylhydrazine in refluxing methanol. Moreover, the presence of a small amount of acetone in the solvents led the isolation of azines **168**, formed from the reaction of hydrazine moiety of **167** (R^2^ = H) with the acetone carbonyl group. Cleavage of the hydrazine moiety of compounds **168** also occurs in acidic medium to provide the respective pyrazoles **169** (Scheme 57) [112].

**Scheme 58:**
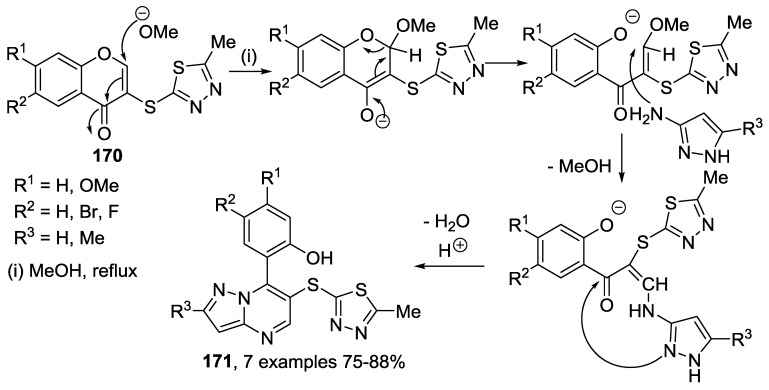
Mechanism of the reaction of 3-[(5-methyl-1,3,4-thiadiazol-2-yl)sulfanyl]chromones **170** with 3(5)-aminopyrazoles in the presence of sodium methoxide in refluxing methanol [113].

Cyclocondensation reaction of 3-[(5-methyl-1,3,4-thiadiazol-2-yl)sulfanyl]chromones **170** with 3(5)-aminopyrazoles in the presence of sodium methoxide in refluxing methanol gave the corresponding 2-{6-[(5-methyl-1,3,4-thiadiazol-2-yl)sulfanyl]pyrazolo[1,5-*a*]pyrimidin-7-yl}phenols **171** in high yields (Scheme 58) [113]. The mechanism proposed involves chromone ring-opening in the presence of base, attack of the amino group of the pyrazoloamine to the C-β carbon (relatively to C=O) and finally, condensation of the pyrazole ring with the carbonyl carbon.

**Scheme 59:**
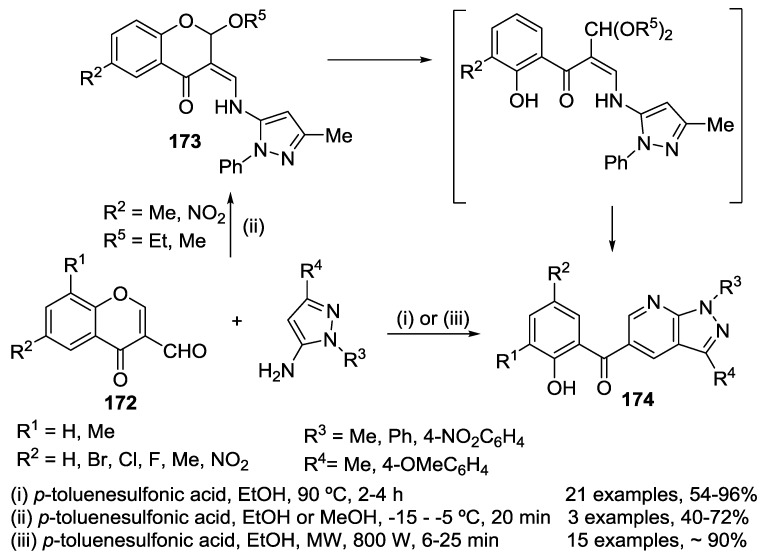
Synthesis of 5-(2-hydroxyaroyl)-1-methyl/phenyl-3-substituted-1*H*-pyrazolo[3,4-*b*]pyridines **174** through the reaction of 3-formylchromones **172** with equimolar amounts of 5-amino-1-methyl/phenyl-3-substituted-1*H*-pyrazoles in the presence of a catalytic amount of *p*-toluenesulfonic acid [114,115,116].

Various 5-(2-hydroxyaroyl)-1-methyl/phenyl-3-substituted-1*H*-pyrazolo[3,4-*b*]pyridines **174** arose from the one-pot reaction of 3-formylchromones **172** with equimolar amounts of 5-amino-1-methyl/phenyl-3-substituted-1*H*-pyrazoles in the presence of a catalytic amount of *p*-toluenesulfonic acid in refluxing ethanol (Scheme 59) [114,115,116]. In fact, Lácová and coworkers also isolated enamine-intermediates 2-alkoxy-6-substituted-3-(3-methyl-1-phenylpyrazol-5-ylaminomethylene)chromanones **173**, when the reaction was performed at low temperature, which helps to explain the formation of the final products [115,116]. This synthesis under microwave irradiation proceeded significantly faster (6–25 min, 800 W) than in classical conditions (2–4 h) and produced clean products in high yields (about 90%). However, the isolation of intermediates **173** was not possible in these conditions [115]. Five pyrazolo[3,4-*b*]pyridines **174** were assessed for their anti-inflammatory activity against tumor necrosis factor-alpha (TNF-α) and interleukin-6 (IL-6). The results showed a 34–60% inhibition at 10 μM concentration against IL-6 (94% of inhibition for the positive control dexamethasone at 1 μM concentration) while none of the compounds showed significant TNF-α inhibitory activity [114].

**Scheme 60:**
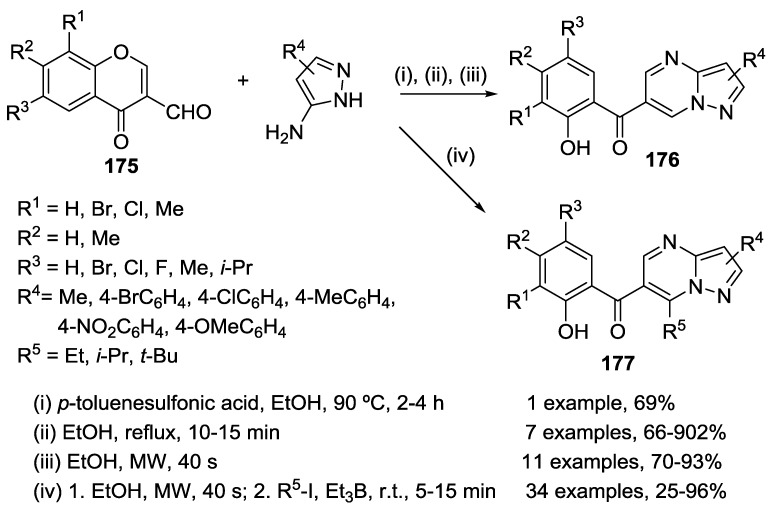
Synthesis of 6-(2-hydroxyaroyl)pyrazolo[1,5-*a*]pyrimidines **176** and **177** through the reaction of 3-formylchromones **175** with 3(5)-aminopyrazoles in different reaction conditions [114,117,118].

**Scheme 61:**
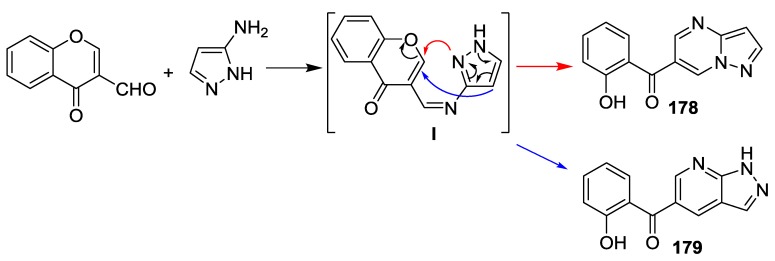
Proposed mechanism for the synthesis of 6-(2-hydroxybenzoyl)pyrazolo[1,5-*a*]pyrimidines **178** or 5-(2-hydroxybenzoyl)-1*H*-pyrazolo[3,4-*b*]pyridines **179** [117].

On the other hand, the reaction of 3-formyl-6-methylchromone **175** with an equimolar amount of 3(5)-amino-5(3)-(4-methylphenyl)-1*H*-pyrazole in the presence of a catalytic amount of *p*-toluenesulfonic acid in refluxing ethanol provided the regioisomer 6-(2-hydroxy-5-methylbenzoyl)-2-(4-methylphenyl)pyrazolo[1,5-*a*]pyrimidine **176** in 69% yield (Scheme 60) [114]. These type of pyrazoles **176** were also obtained from the reaction of 3-formylchromones with equimolar amounts of 3(5)-amino-5(3)-substituted pyrazoles in ethanol at reflux [117] or under microwave irradiation [118] (Scheme 60). The mechanism proposed for the formation of regioisomers **178** and **179** involves the condensation between the amino group at the pyrazole unit and the aldehyde group at the chromone ring to give intermediates **I** (Scheme 61). Then, intermediate **I** can follow an intramolecular ring opening of the chromone ring though nucleophilic displacement by attack of the nucleophilic nitrogen at the pyrazole ring to compound **178**. The alternative is by attack of the C-4 at the pyrazole instead of the nitrogen to give regioisomer **179** [117]. To note that Zimmerman and coworkers also studied the two-step one-pot tandem reaction of 3-formylchromones **175** with equimolar amounts of 3(5)-amino-5(3)-substituted pyrazoles, via microwave-assisted protocol, to give the corresponding pyrazolo-pyrimidines, which underwent intermolecular radical addition in the presence of alkyliodides and triethylborane providing the substituted pyrazolopyrimidines **177** (Scheme 60) [118].

**Scheme 62:**
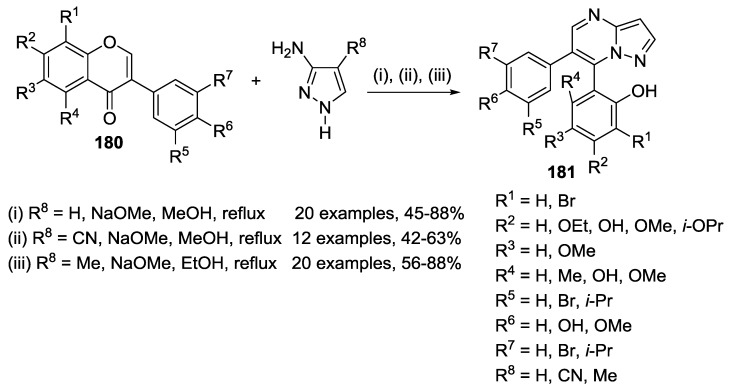
Reaction of 3-arylchromones **180** with 3(5)-aminopyrazoles to afford 6,7-diarylpyrazolo[1,5-*a*]pyrimidines **181** [119,120,121].

A wide range of 6,7-diarylpyrazolo[1,5-*a*]pyrimidines **181** have been efficiently synthesized through the reaction of various 3-arylchromones **180** with 3(5)-aminopyrazoles in the presence of sodium methoxide in refluxing methanol [119,120] or ethanol [121] (Scheme 62). Under microwave irradiation, the reaction of 6,7-disubstituted chromones with 3-aminopyrazoles and sodium methoxide in dried DMSO gave a mixture of 5- and 7-(2-hydroxyaryl)pyrazolo[1,5-*a*]pyrimidines, being the 5-isomer the most abundant one [122]. Moreover, electron-donating groups in the chromone moiety provided better overall yields than those presenting electron-withdrawing groups. The antifungal activity of both isomers were screened against five phytopathogenic fungi (*Cytospora* sp., *Colletotrichum gloeosporioides*, *Botrytis cinerea*, *Alternaria solani* and *Fusarium solani*). All compounds exhibited antifungal activities against these five fungi strains in different levels and two of them possess high antifungal abilities against *Colletotrichum gloeosporioides* with the IC_50_ values of 24.90 and 28.28 μg/mL, respectively (the positive control hymexazol present an IC_50_ value >100 μg/mL) [122].

**Scheme 63:**
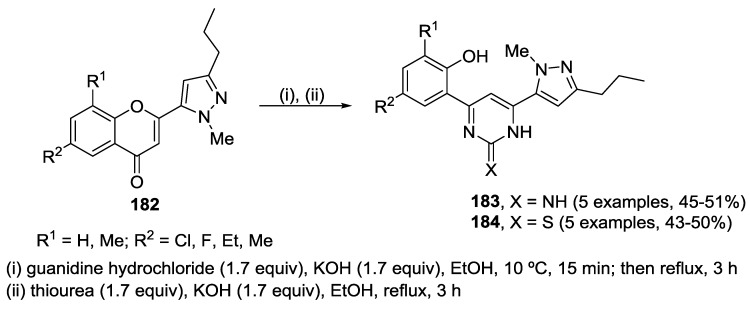
Transformation of 2-substituted chromone-pyrazole dyads **182** into the corresponding disubstituted 1,2-dihydro-2-iminopyrimidines **183** and 1,2-dihydro-2-pyrimidinethiones **184** [8].

The reaction of 2-(1,3-disubstituted pyrazolyl)chromones **182** with guanidine hydrochloride and thiourea in the presence of potassium hydroxide in ethanol afforded 6-[2-imino-6-(1-methyl-3-propyl-1*H*-5-pyrazolyl)-1,2-dihydro-4-pyrimidinyl]phenols **183** (45–51%) and 4-(2-hydroxyphenyl)-6-(1-methyl-3-propyl-1*H*-5-pyrazolyl)-1,2-dihydro-2-pyrimidinethiones **184** (43–50%), respectively (Scheme 63) [8].

## 6. Conclusions

In this review we have presented several strategies that have been developed, since the beginning of the 21st century, towards the synthesis of chromone related pyrazoles, namely chromone-pyrazole dyads, chromone-pyrazole-fused compounds and 3(5)-(2-hydroxyaryl)-pyrazoles, among other pyrazole derivatives. Thus, several chromone-pyrazole dyads have been synthesized, by cyclization of 1,3-dicarbonyl compounds, such as 1,3-diketones, and oxidative cyclization of 2′-hydroxychalcone-type compounds both bearing a pyrazole moiety. Other methods to prepare these dyads include cycloaddition reactions and Knoevenagel-type condensations. Only a few examples of chromone-pyrazole-fused compounds were found. The most straightforward methods to synthesize these compounds include, tandem *O*-arylation-oxidative coupling reactions, cycloaddition reactions and multicomponent reactions. The limited number of examples found suggests that the synthesis of this type of compounds deserves greater attention from synthetic chemists. A huge number of 3(5)-(2-hydroxyaryl)pyrazoles have been synthesized through the reaction of several chromone derivatives with hydrazines in varied experimental conditions. Also a wide variety of 3-formylchromones were found to react with aminopyrazoles giving pyrazole-pyridines and pyrazole-pyrimidines containing a 2-hydroxyaroyl moiety in their structures. The transformations presented in this review led to a huge variety of compounds possessing both nitrogen and oxygen heterocycles. The comprehensive details of these transformations and several mechanistic considerations were also presented.
